# Supplementary material for: Identification of Transcription Factors Regulating SARS-CoV-2 Tropism Factor Expression by Inferring Cell-Type-Specific Transcriptional Regulatory Networks in Human Lungs
Source: Viruses. 2022 Apr 17;14(4):837. doi: 10.3390/v14040837 (PMC9026071; doi:10.3390/v14040837)
Supplement: Supplementary file 1 [file viruses-14-00837-s001.zip › Figure S13.pdf]

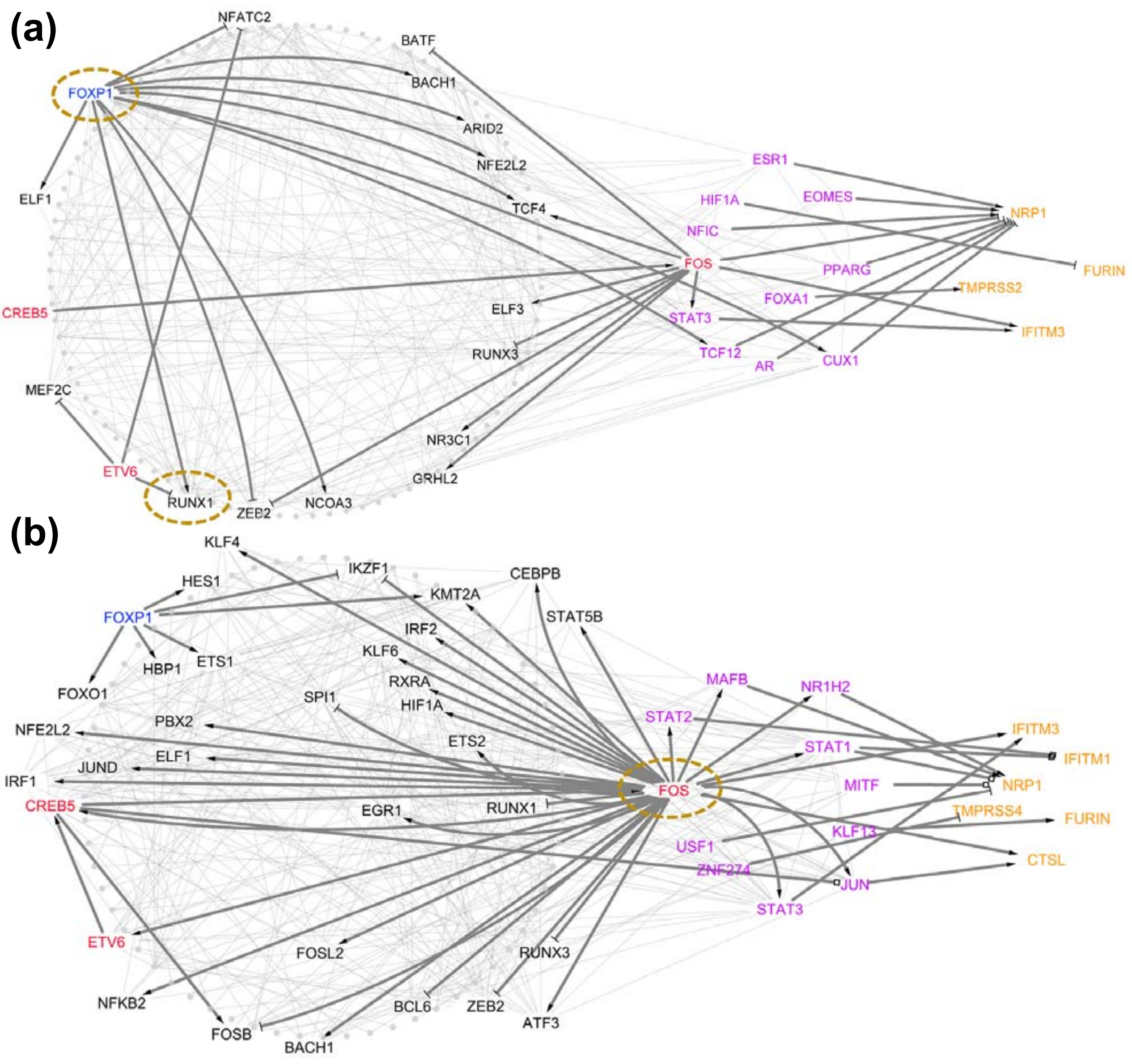

(a)
NFATC2
BATF
FOXP1
BACH1
ARID2
NFE2L2
ESR1
ELF1
TCF4
HIF1A
EOMES
NRP1
NFIC
FOS
PPARG
FURIN
TMPRSS2
CREB5
ELF3
FOXA1
STAT3
IFITM3
RUNX3
TCF12
AR
CUX1
MEF2C
NR3C1
ETV6
GRHL2
RUNX1
ZEB2
NCOA3
(b)
KLF4
IKZF1
CEBPB
HES1
KMT2A
FOXP1
STAT5B
IRF2
KLF6
HBP1
ETS1
MAFB
NR1H2
RXRA
FOXO1
SPI1
HIF1A
STAT2
PBX2
IFITM3
NFE2L2
ETS2
STAT1
ELF1
IFITM1
JUND
IRF1
MITF
NRP1
FOS
CREB5
RUNX1
EGR1
TMPRSS4
FURIN
KLF13
USF1
CTSL
ZNF274
JUN
STAT3
ETV6
FOSL2
RUNX3
NFKB2
BCL6
ZEB2
ATF3
FOSB
BACH1
