# Supplementary figures and images for: Identification of Transcription Factors Regulating SARS-CoV-2 Tropism Factor Expression by Inferring Cell-Type-Specific Transcriptional Regulatory Networks in Human Lungs
Source: Viruses. 2022 Apr 17;14(4):837. doi: 10.3390/v14040837 (PMC9026071; doi:10.3390/v14040837)

**(a)**

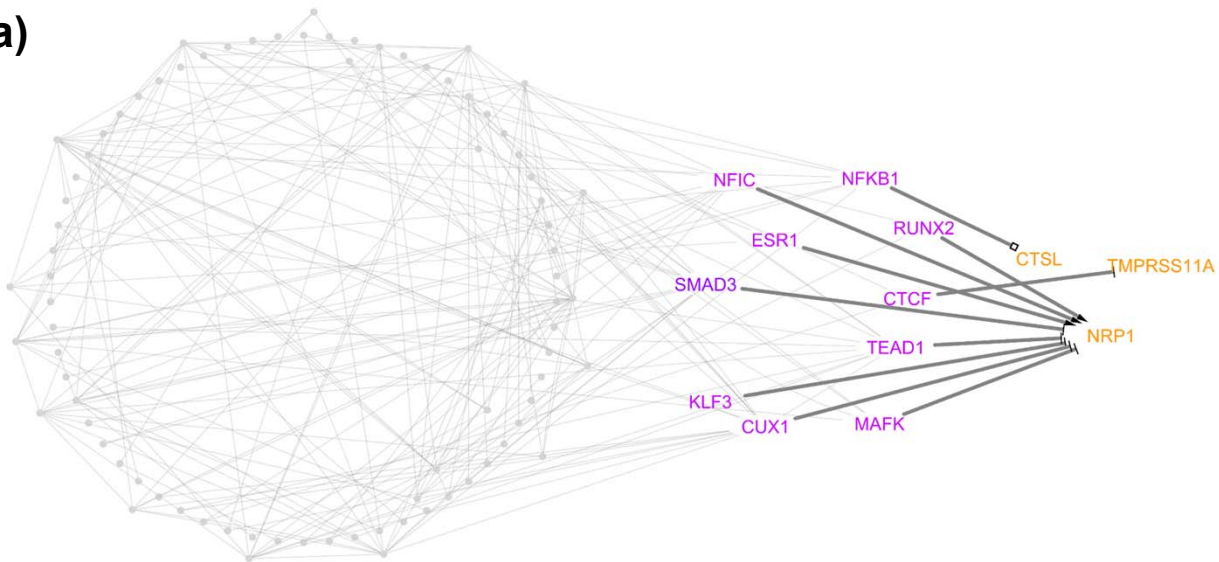

**(b)**

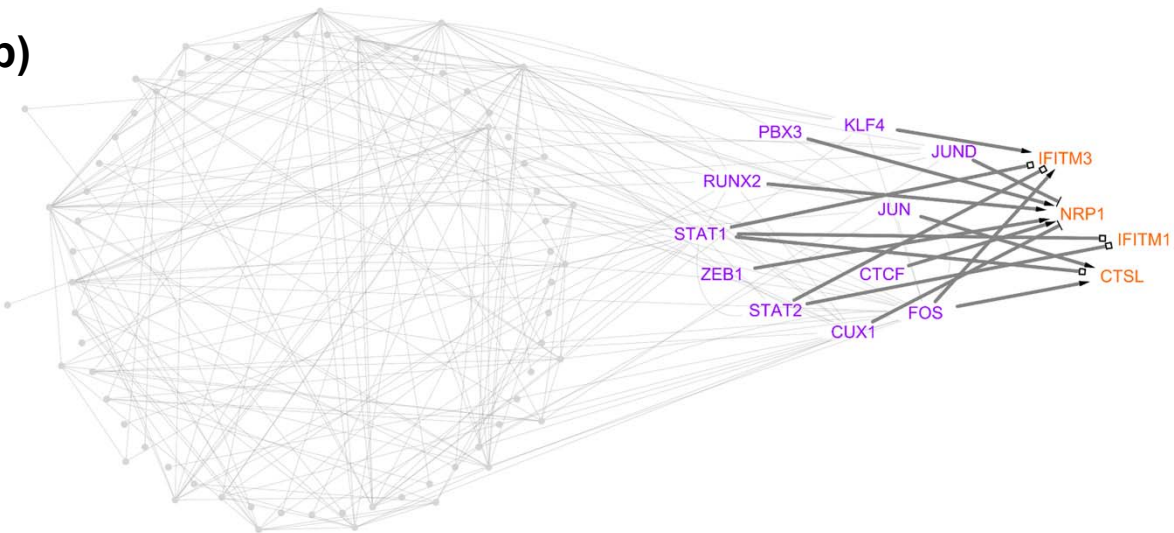

Supplement: Supplementary file 1 [file viruses-14-00837-s001.zip › Figure S1.pdf]

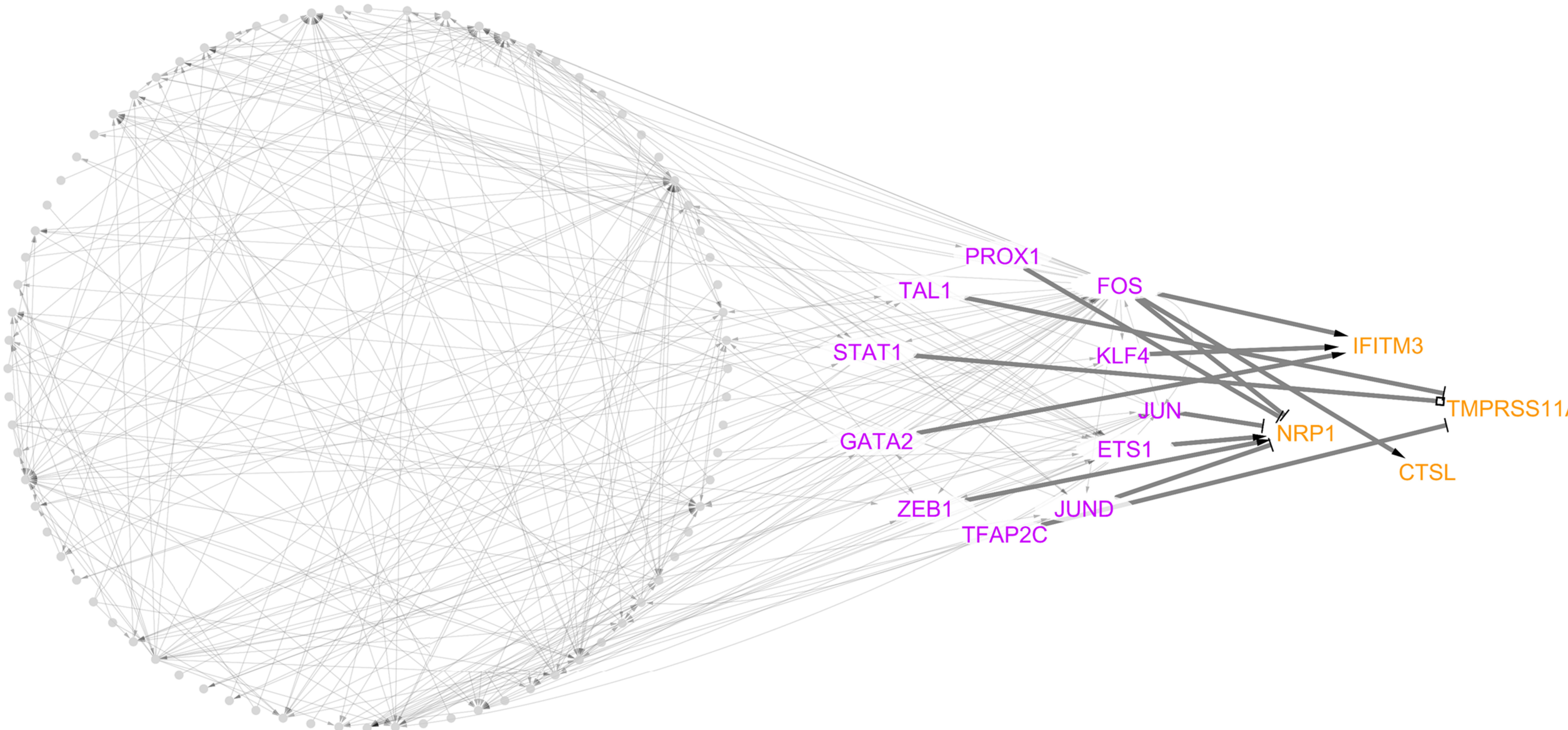

Supplement: Supplementary file 1 [file viruses-14-00837-s001.zip › Figure S10.pdf]

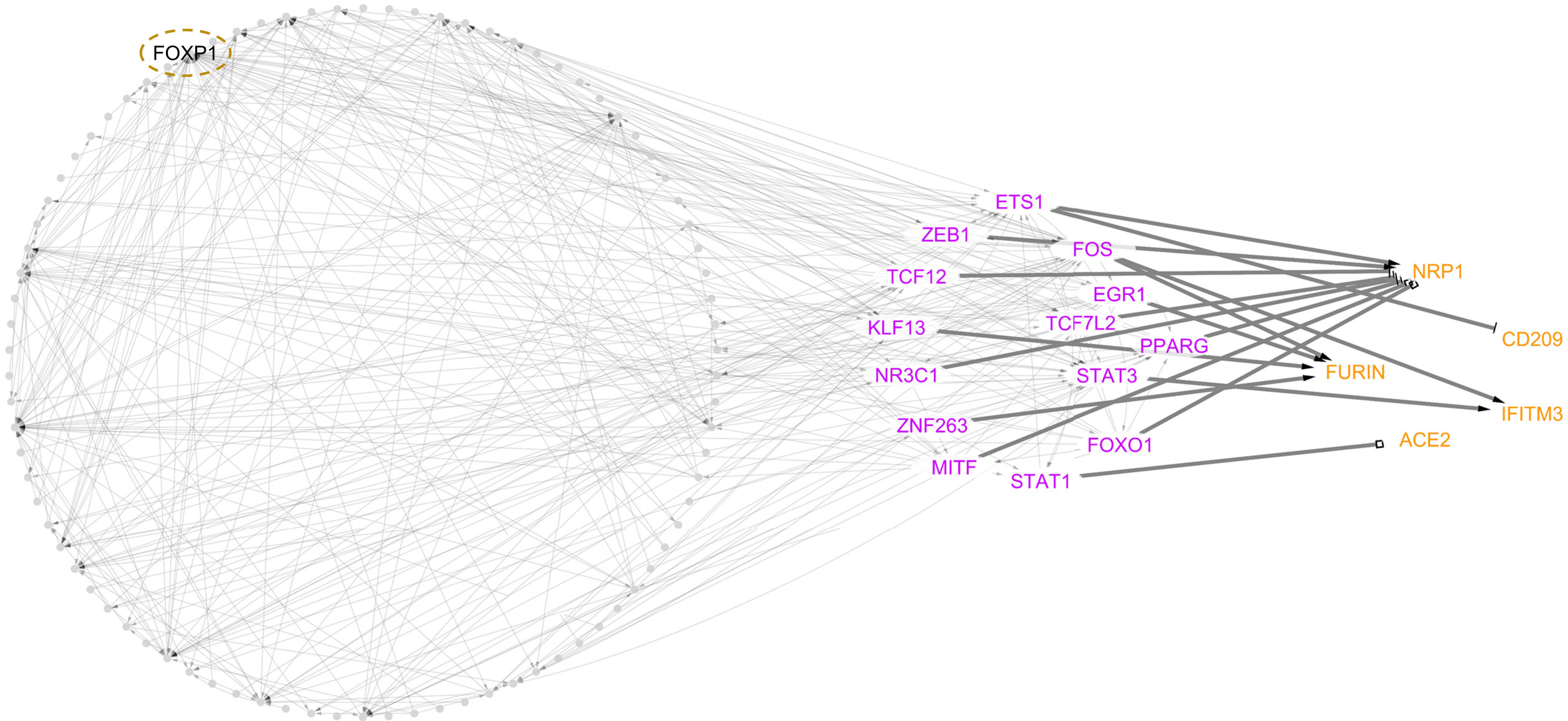

Supplement: Supplementary file 1 [file viruses-14-00837-s001.zip › Figure S11.pdf]

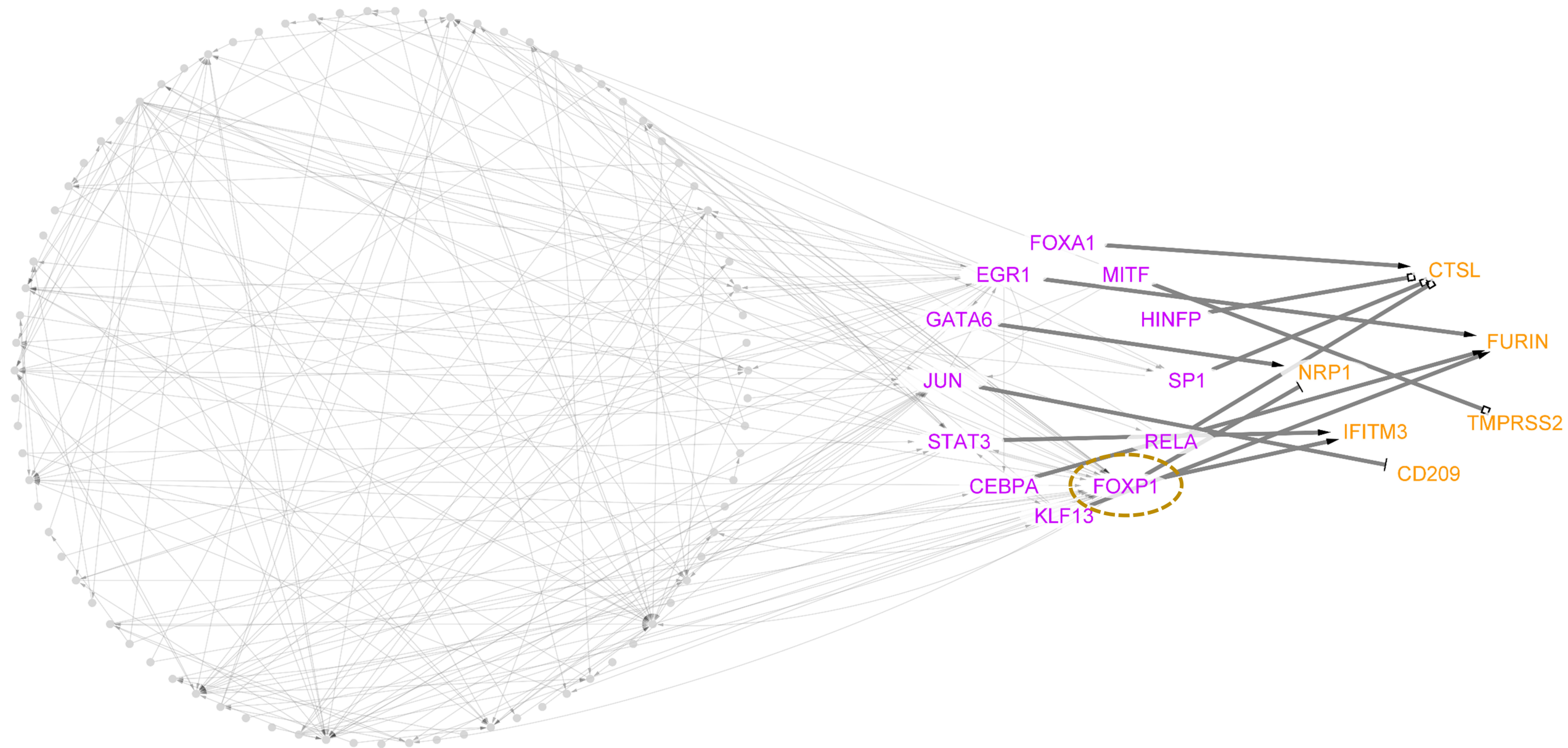

Supplement: Supplementary file 1 [file viruses-14-00837-s001.zip › Figure S12.pdf]

**(a)**

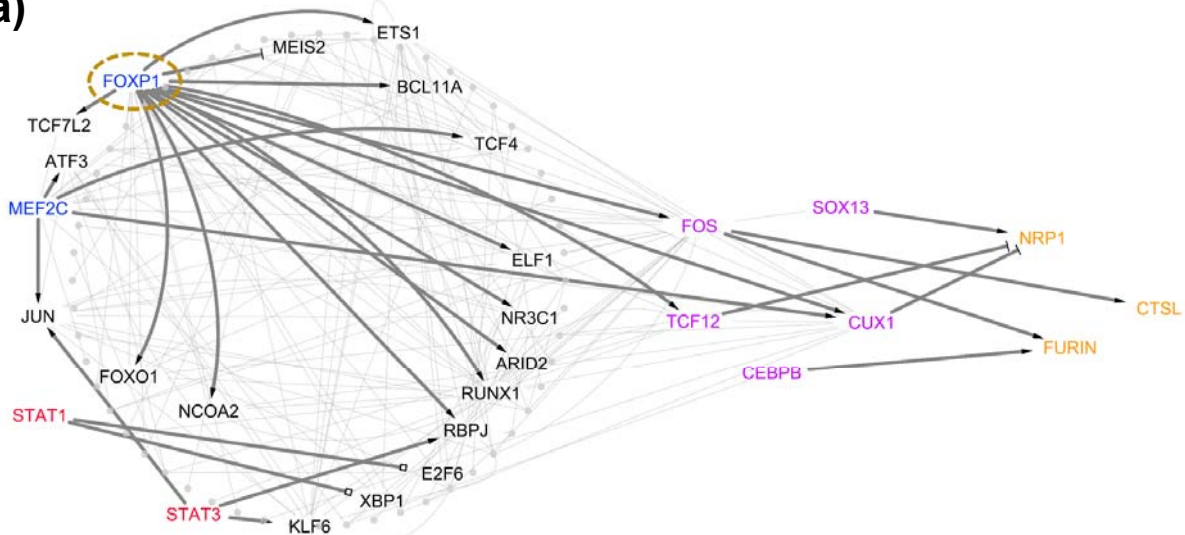

**(b)**

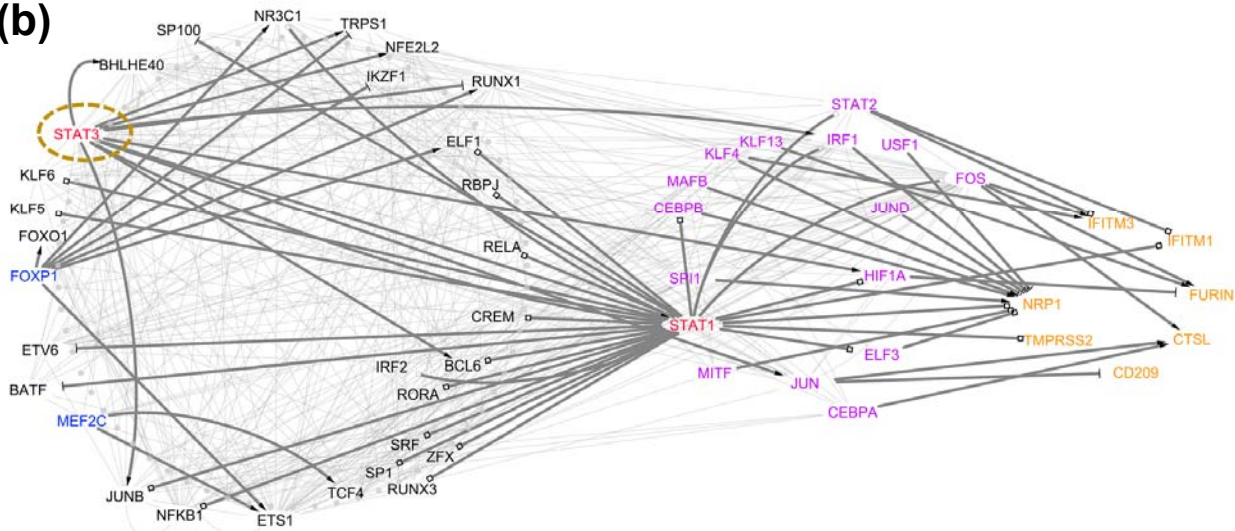

Supplement: Supplementary file 1 [file viruses-14-00837-s001.zip › Figure S14.pdf]

**(a)**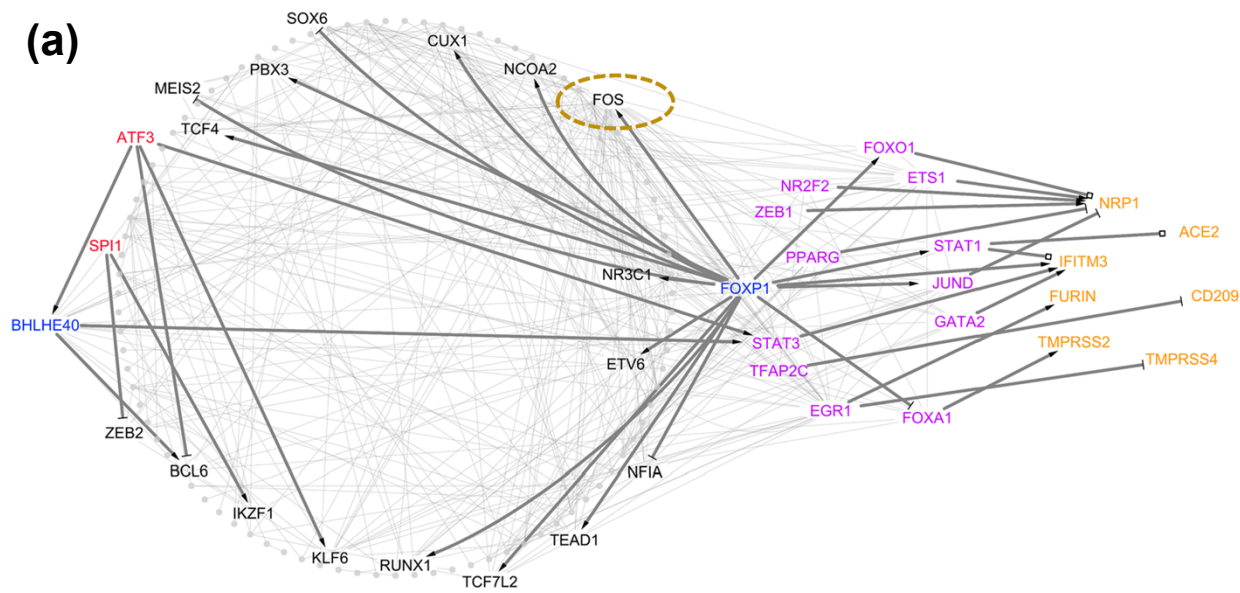**(b)**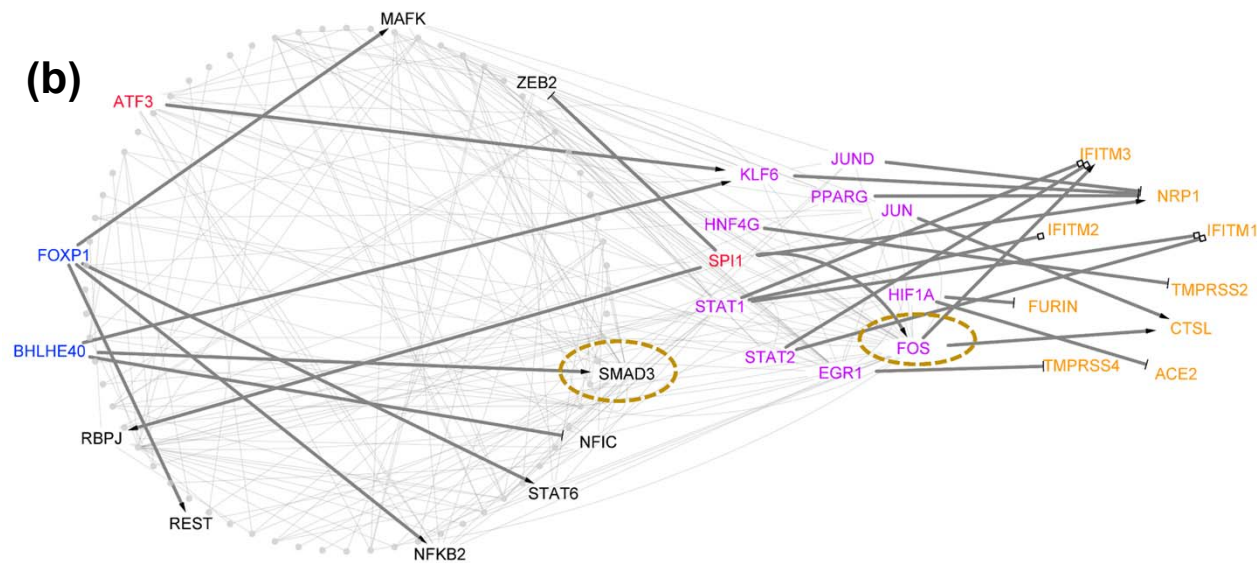

Supplement: Supplementary file 1 [file viruses-14-00837-s001.zip › Figure S15.pdf]

**(a)**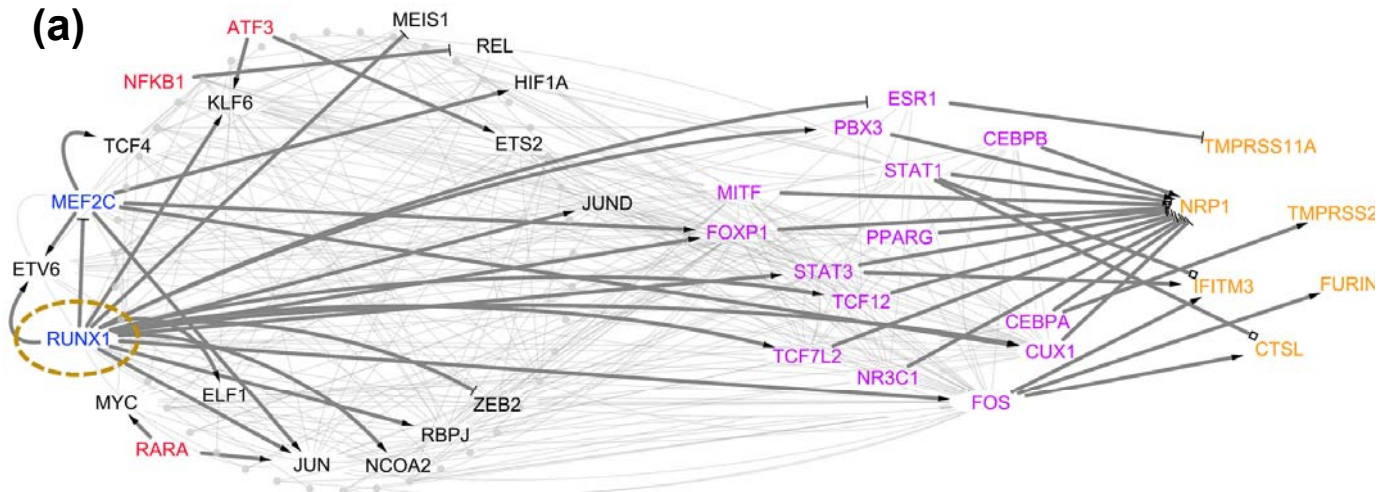**(b)**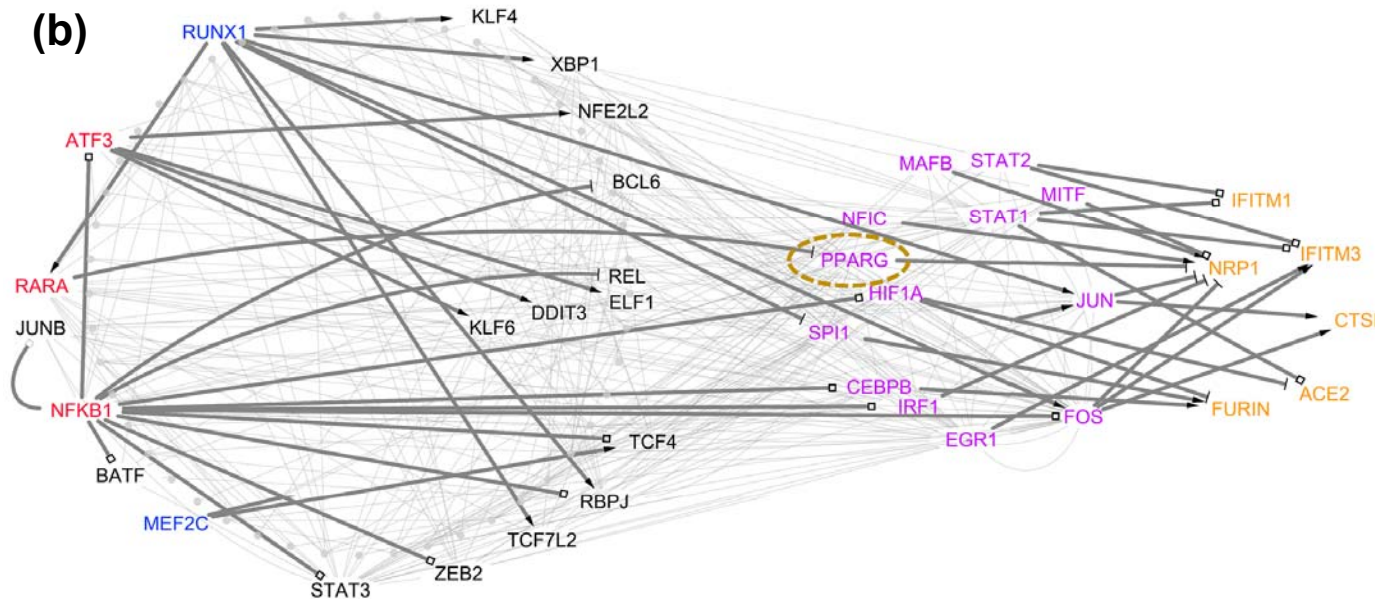

Supplement: Supplementary file 1 [file viruses-14-00837-s001.zip › Figure S17.pdf]

**(a)**

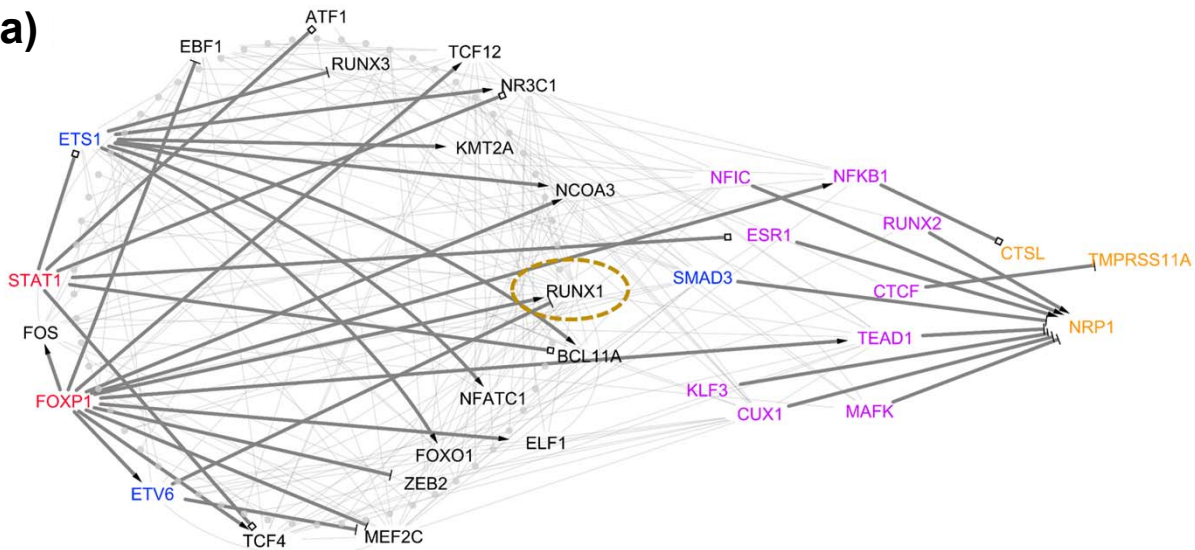

**(b)**

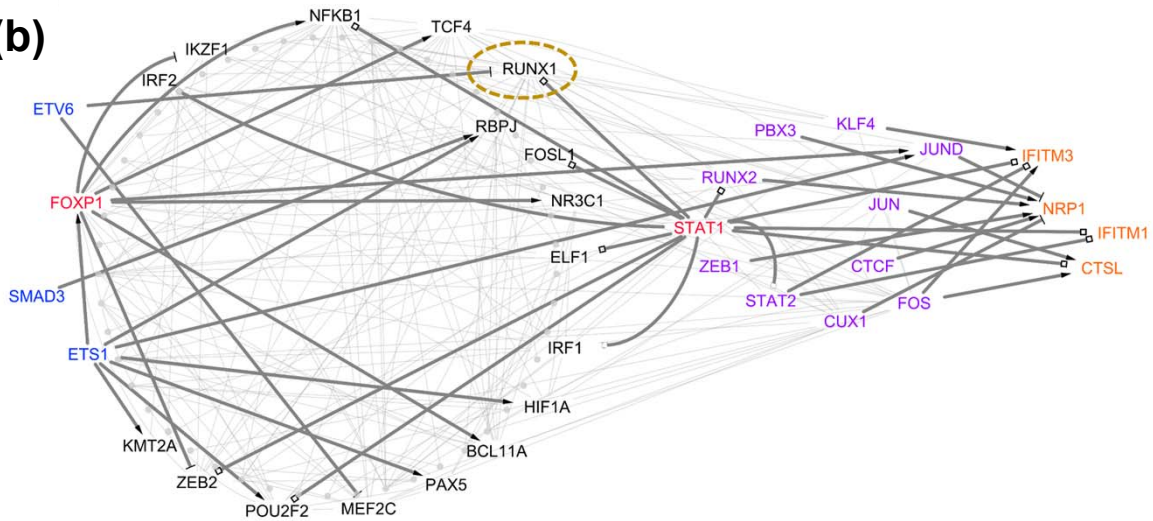

Supplement: Supplementary file 1 [file viruses-14-00837-s001.zip › Figure S18.pdf]

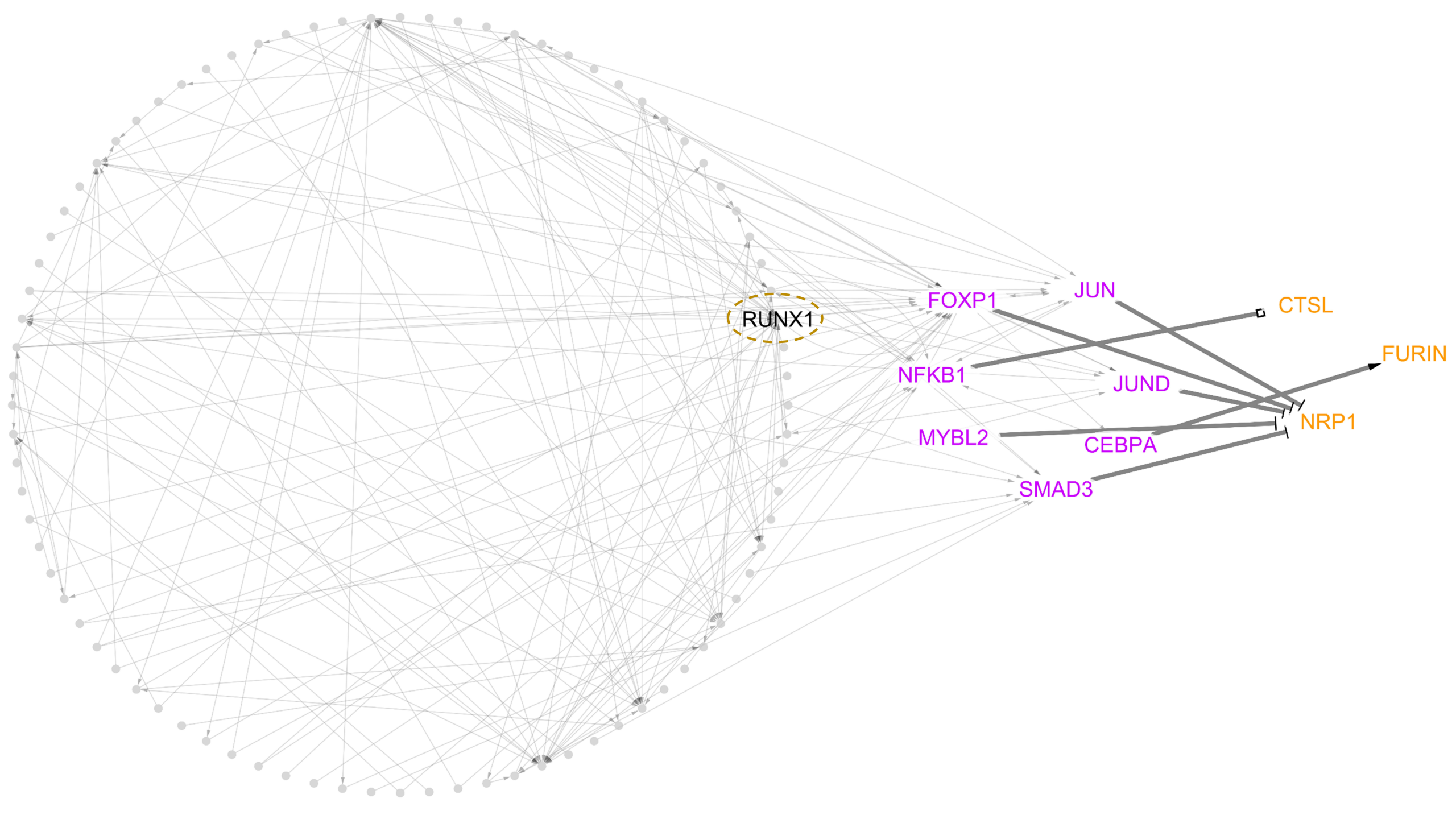

Supplement: Supplementary file 1 [file viruses-14-00837-s001.zip › Figure S19.pdf]

**(a)**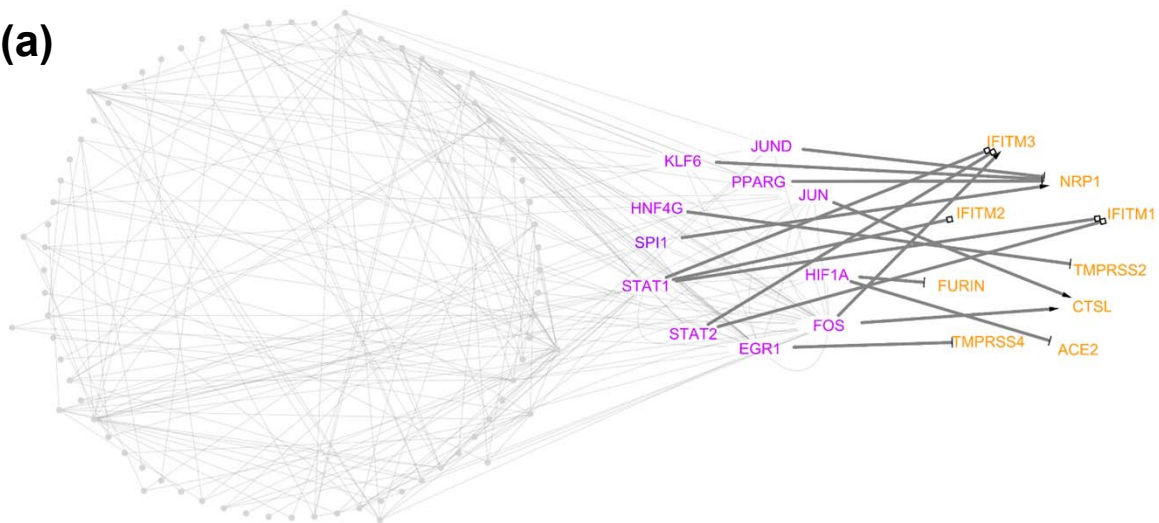**(b)**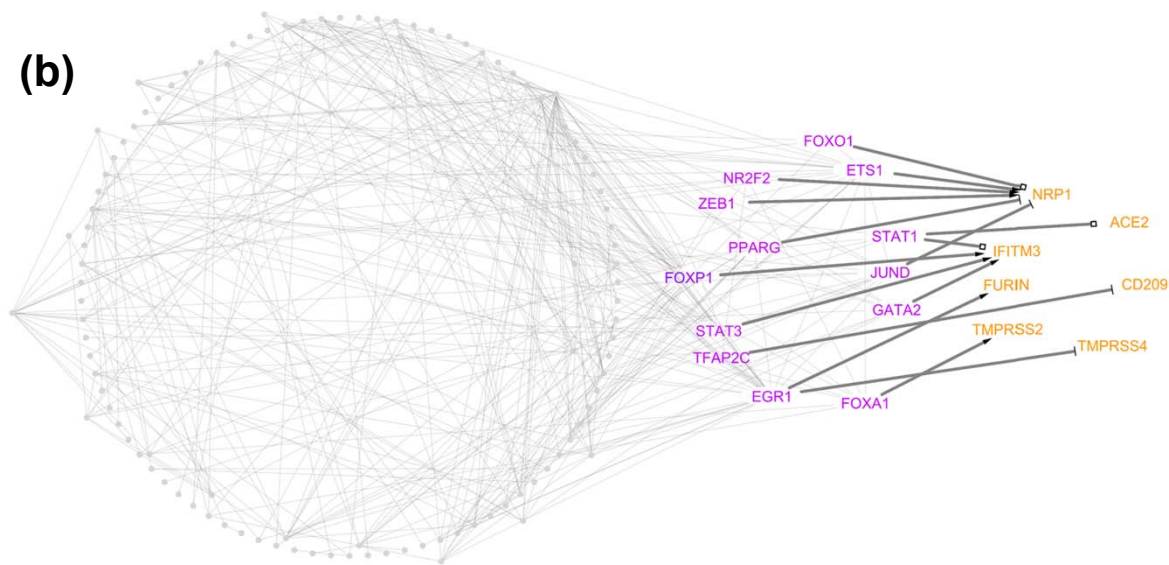

Supplement: Supplementary file 1 [file viruses-14-00837-s001.zip › Figure S2.pdf]

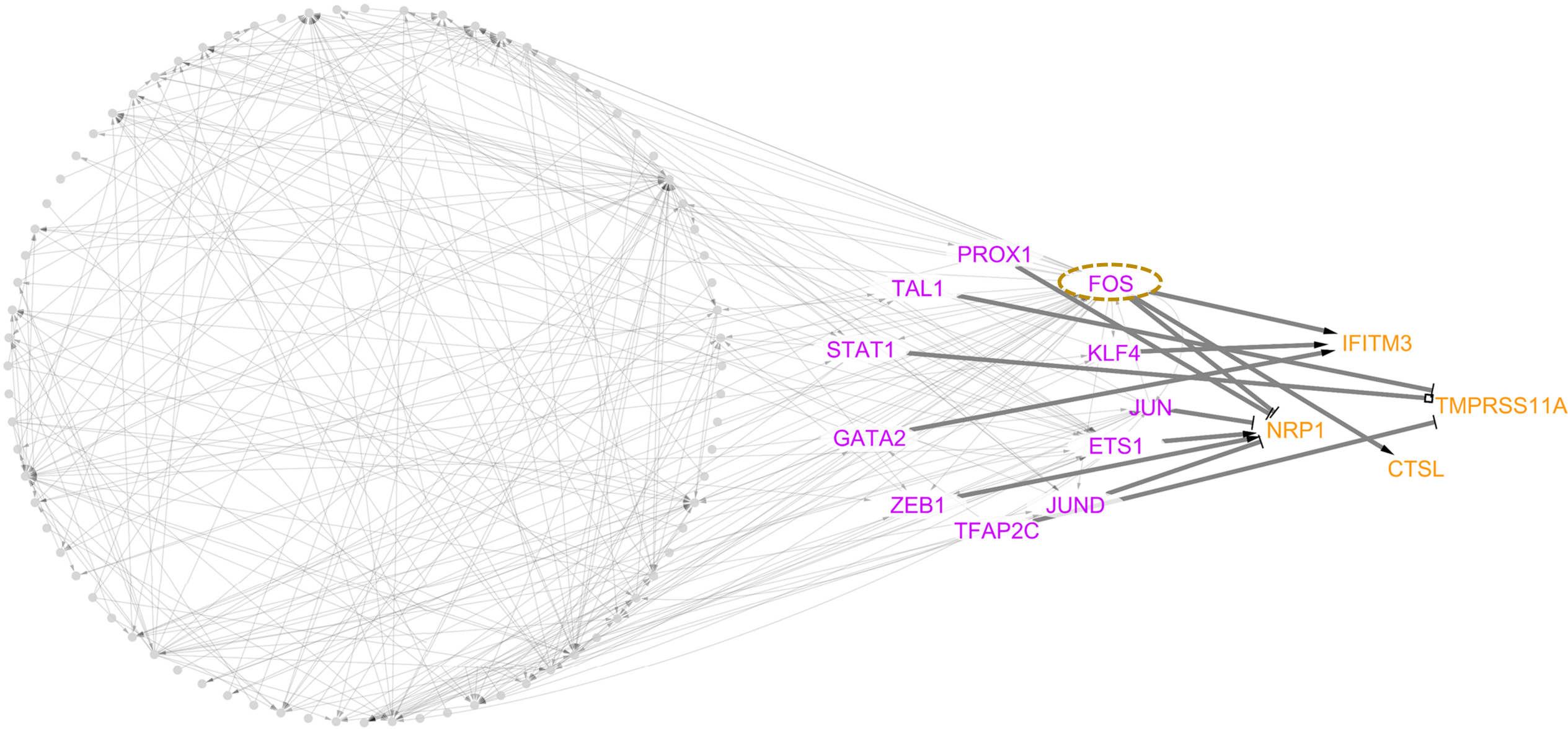

Supplement: Supplementary file 1 [file viruses-14-00837-s001.zip › Figure S20.pdf]

**(a)**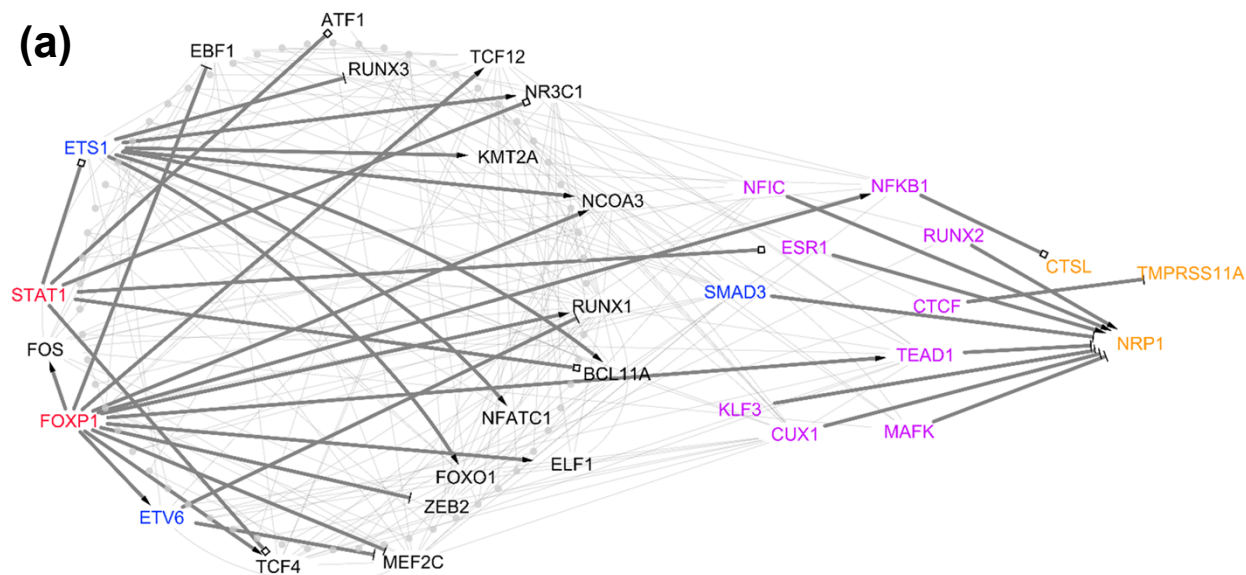**(b)**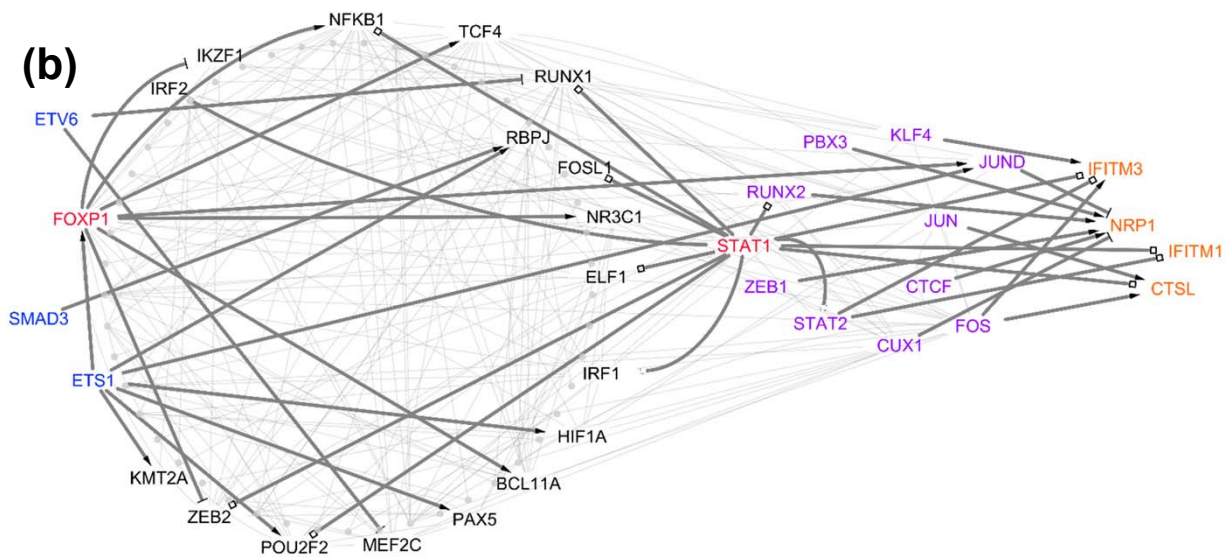

Supplement: Supplementary file 1 [file viruses-14-00837-s001.zip › Figure S21.pdf]

(a)

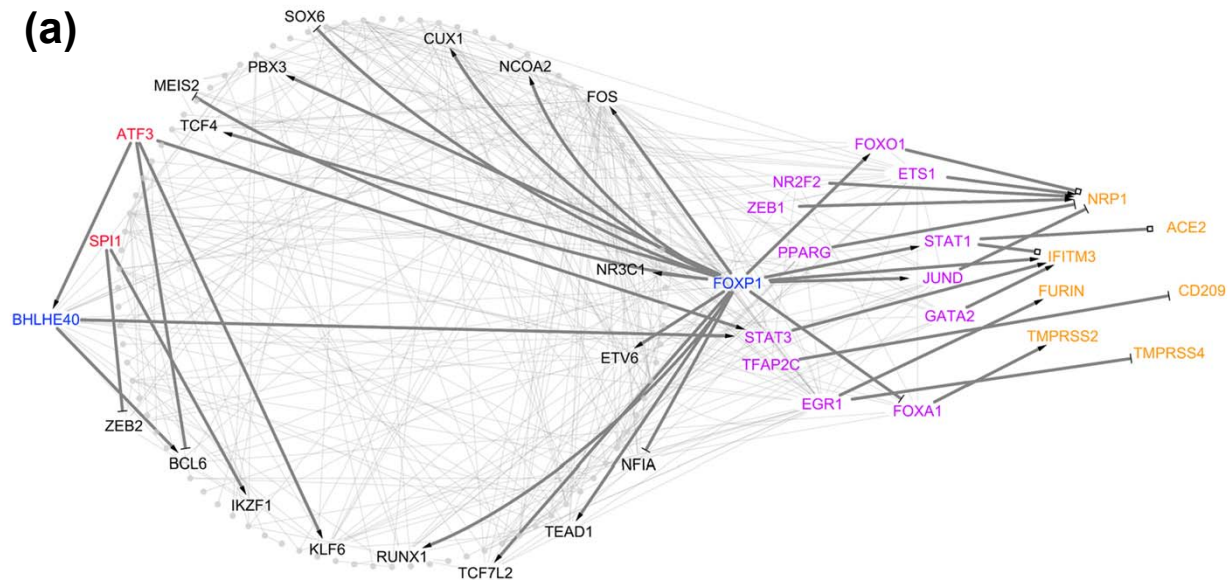

(b)

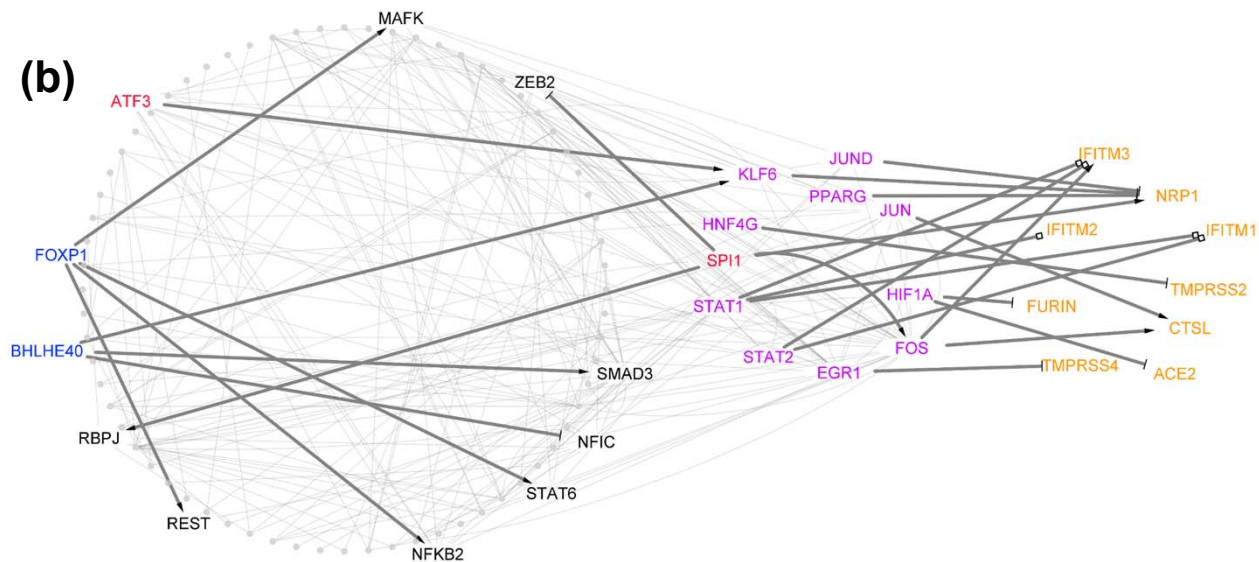

Supplement: Supplementary file 1 [file viruses-14-00837-s001.zip › Figure S22.pdf]

(a)

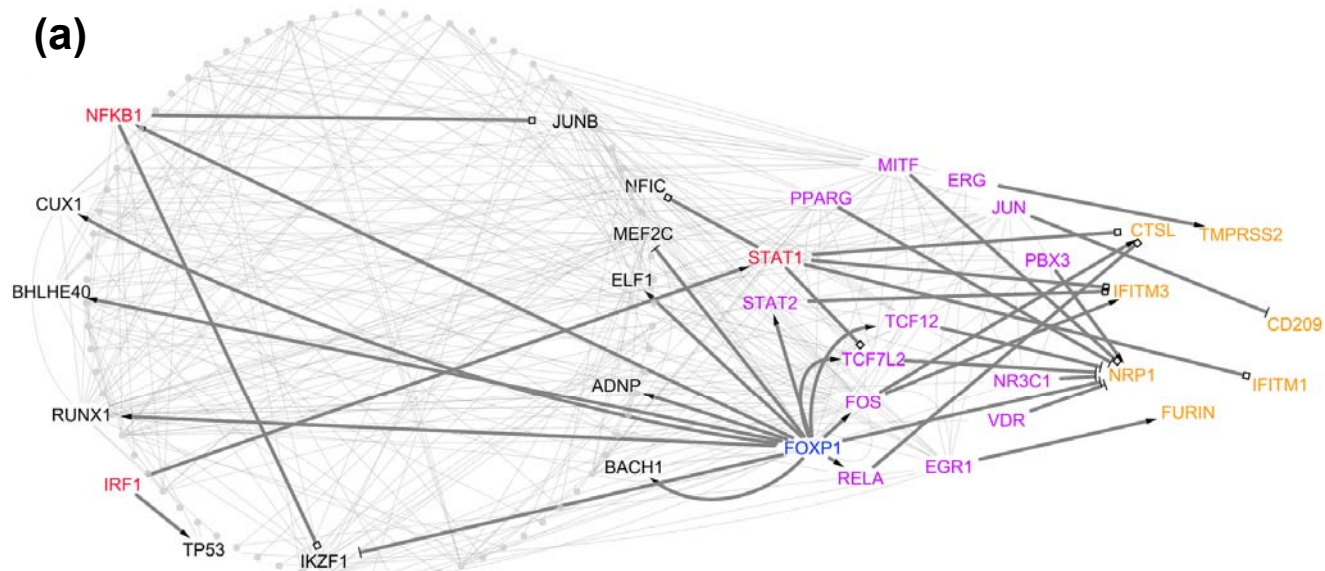

(b)

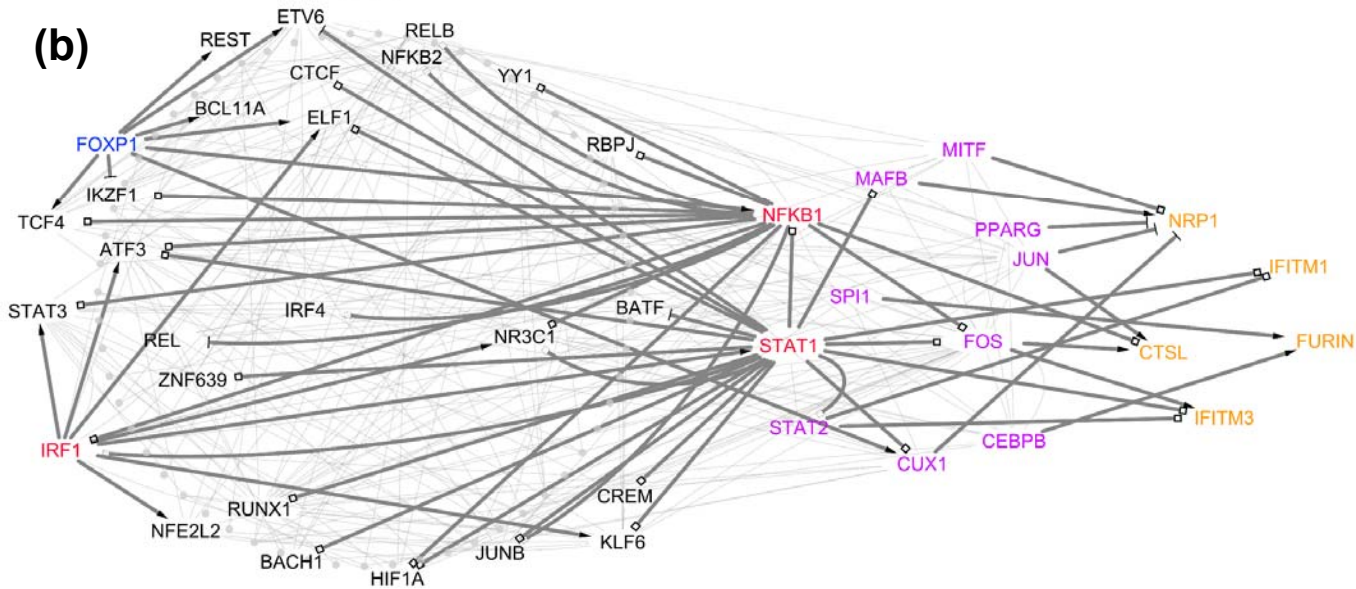

Supplement: Supplementary file 1 [file viruses-14-00837-s001.zip › Figure S23.pdf]

**(a)**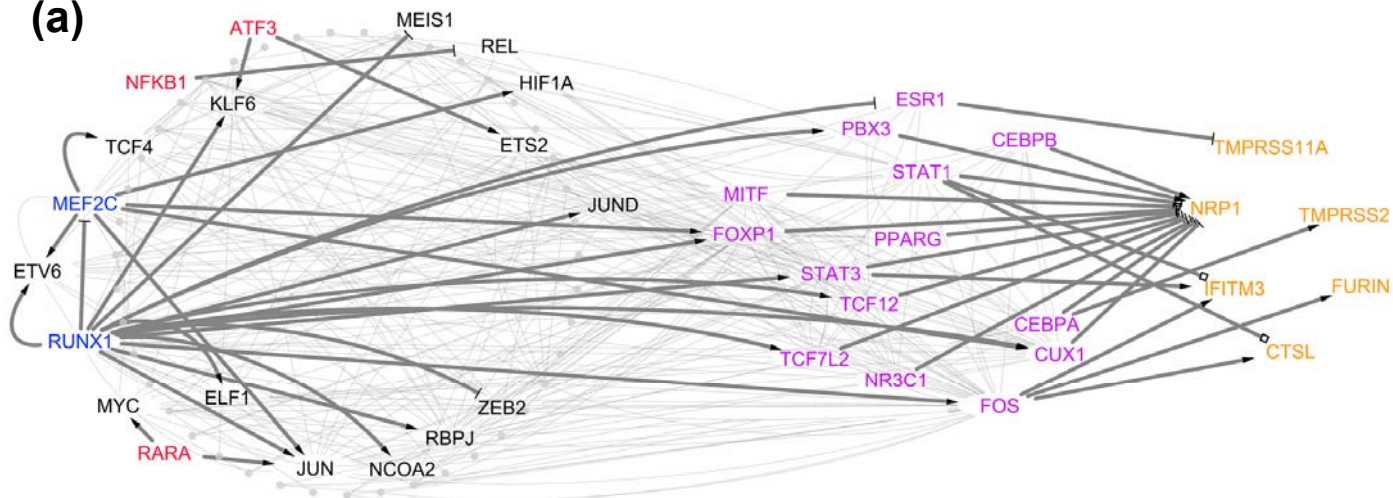**(b)**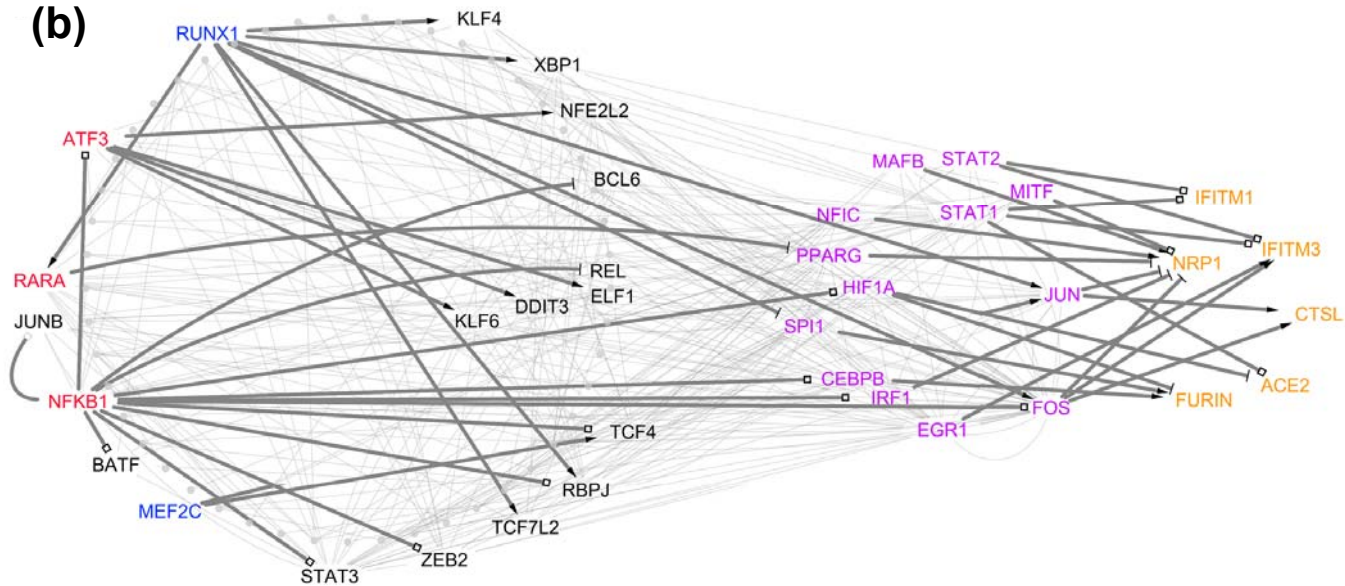

Supplement: Supplementary file 1 [file viruses-14-00837-s001.zip › Figure S24.pdf]

**(a)**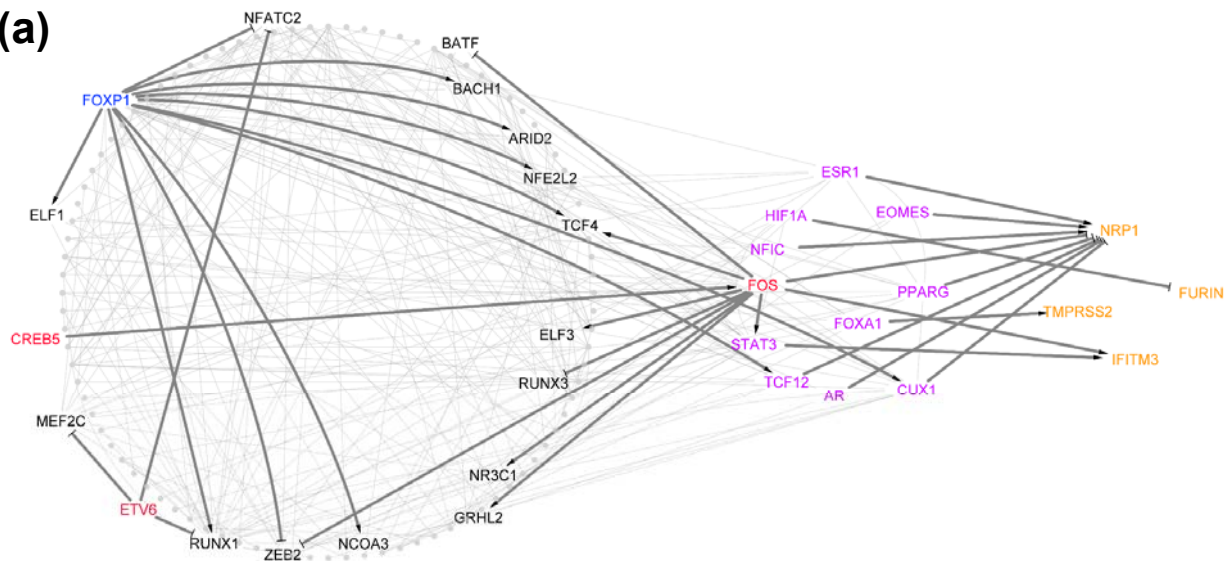**(b)**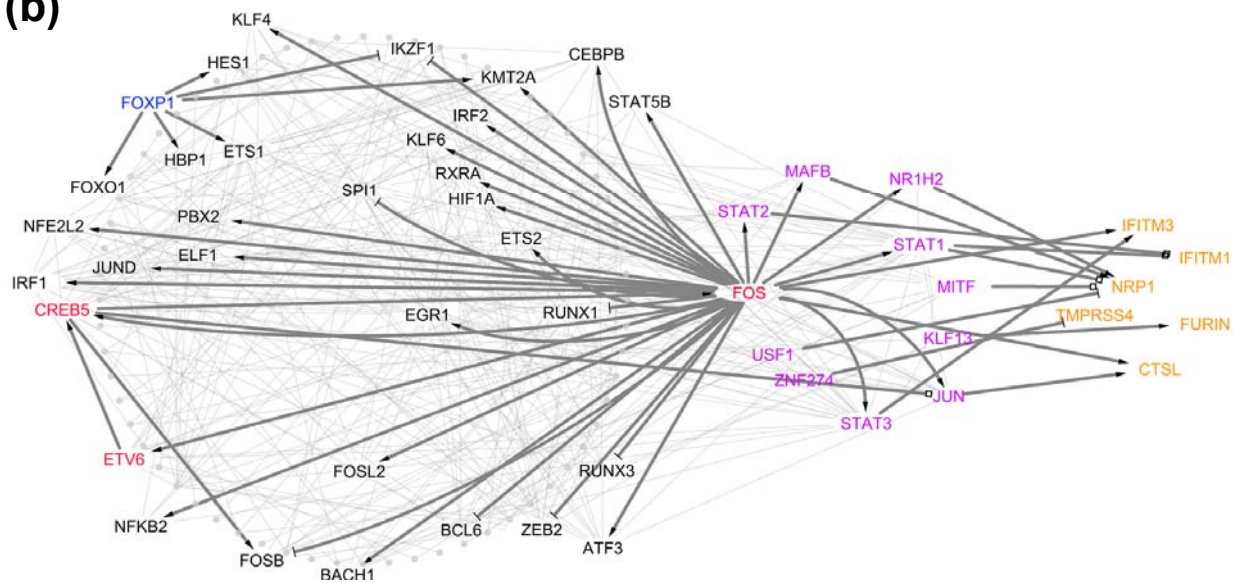

Supplement: Supplementary file 1 [file viruses-14-00837-s001.zip › Figure S25.pdf]

**(a)**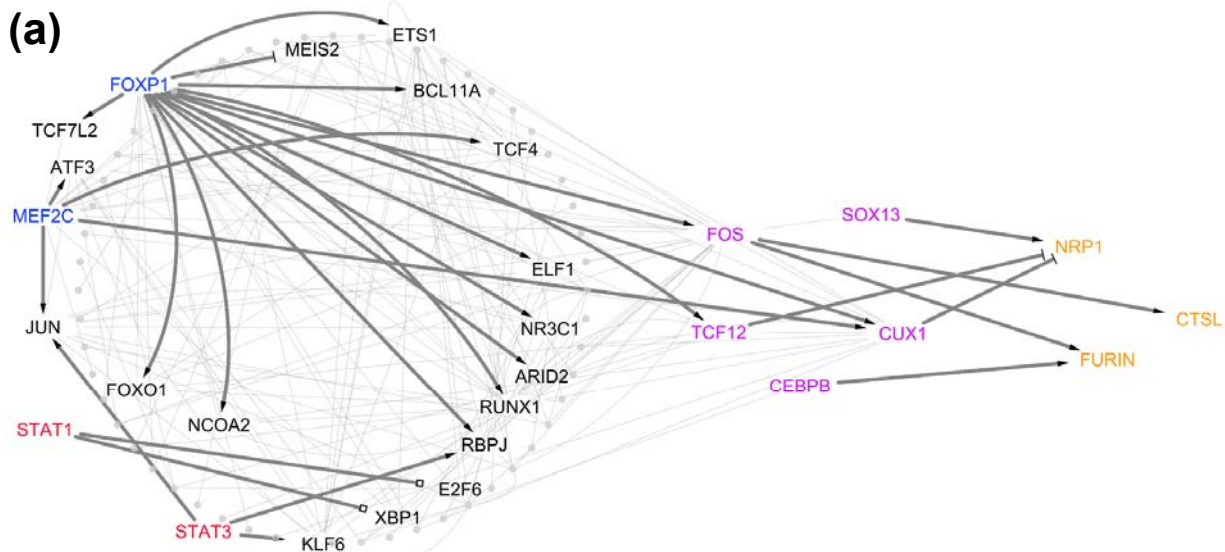**(b)**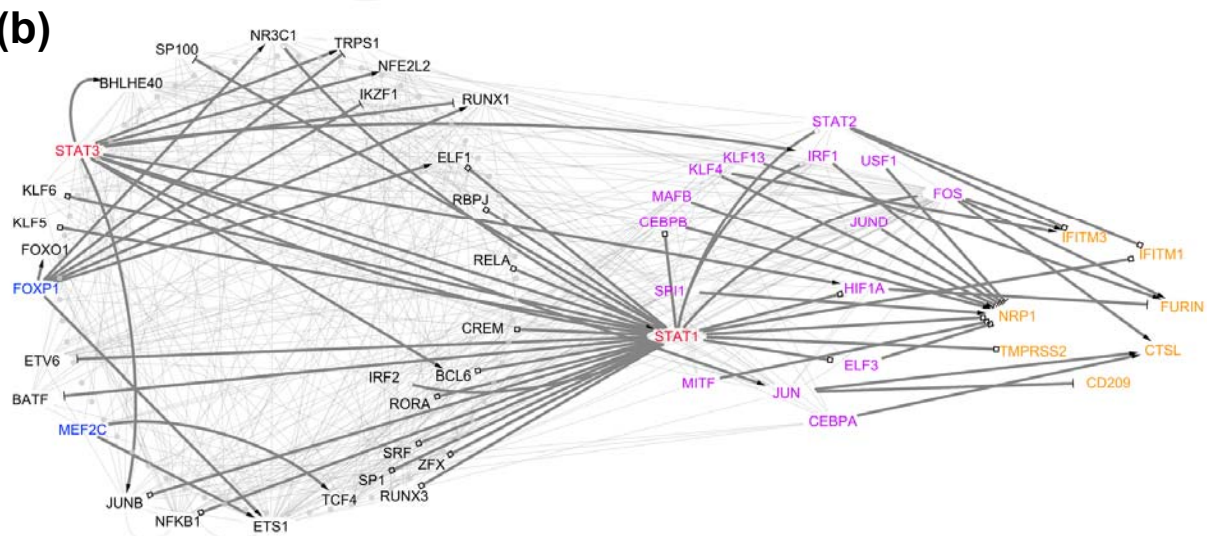

Supplement: Supplementary file 1 [file viruses-14-00837-s001.zip › Figure S26.pdf]

(a)

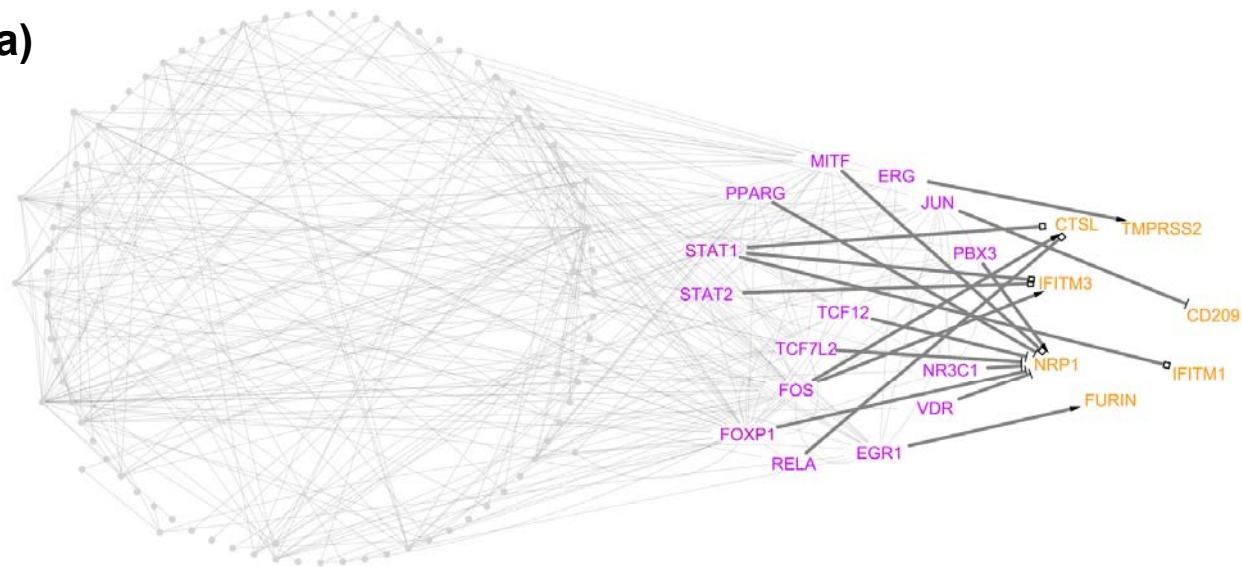

(b)

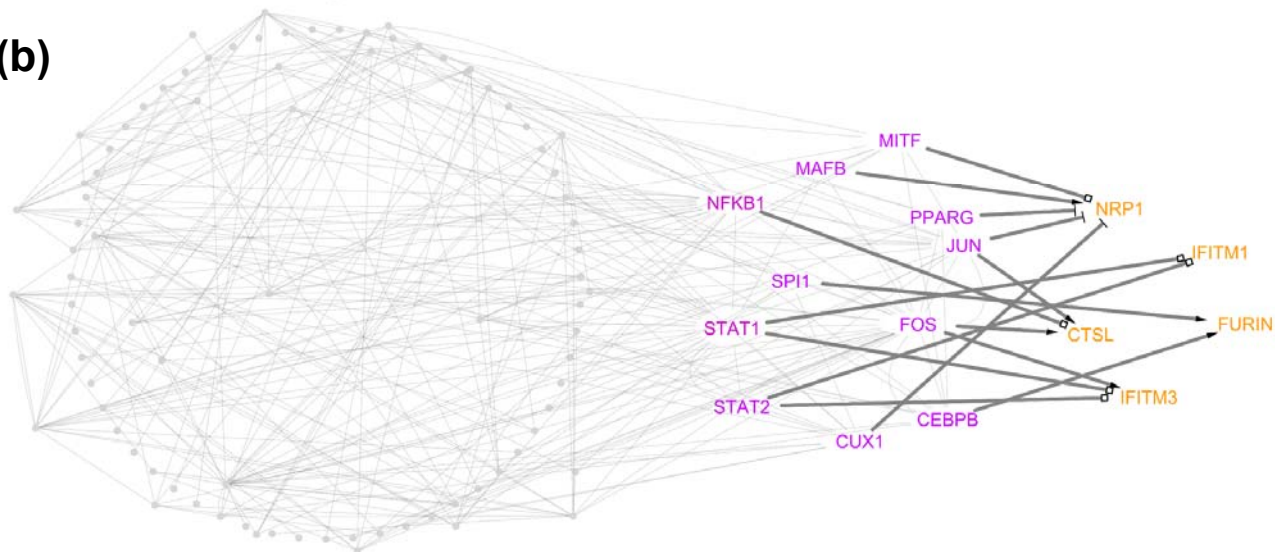

Supplement: Supplementary file 1 [file viruses-14-00837-s001.zip › Figure S3.pdf]

(a)

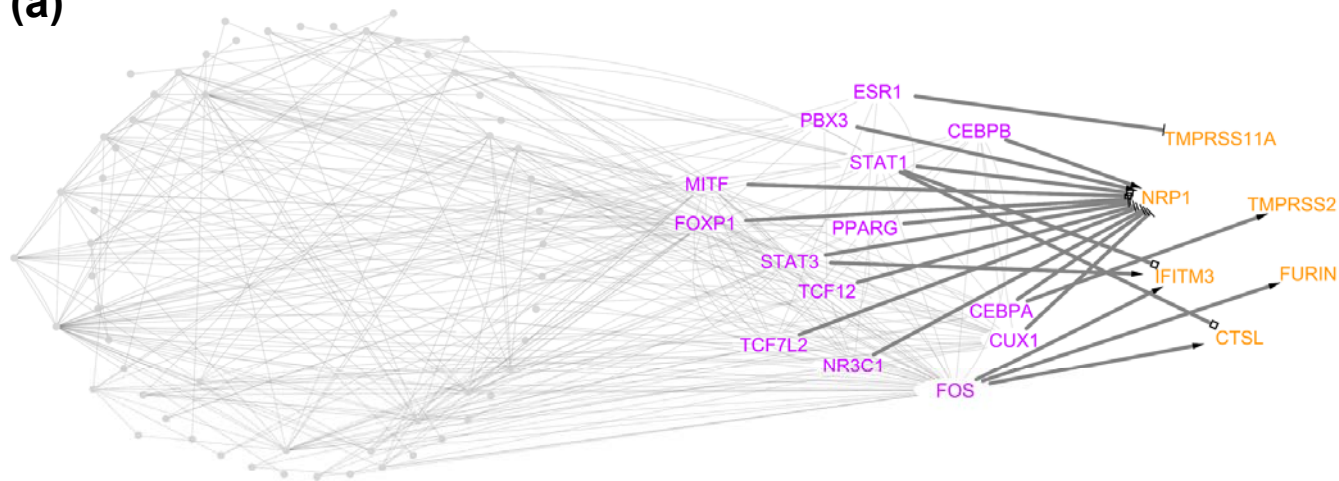

(b)

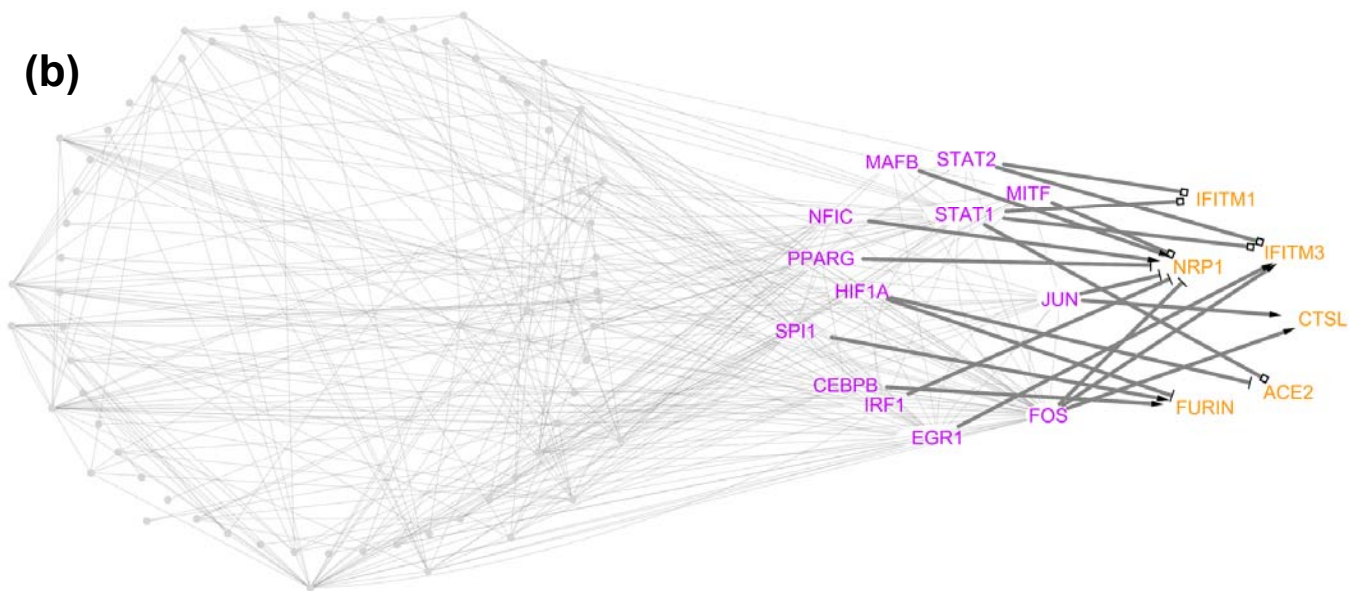

Supplement: Supplementary file 1 [file viruses-14-00837-s001.zip › Figure S4.pdf]

**(a)**

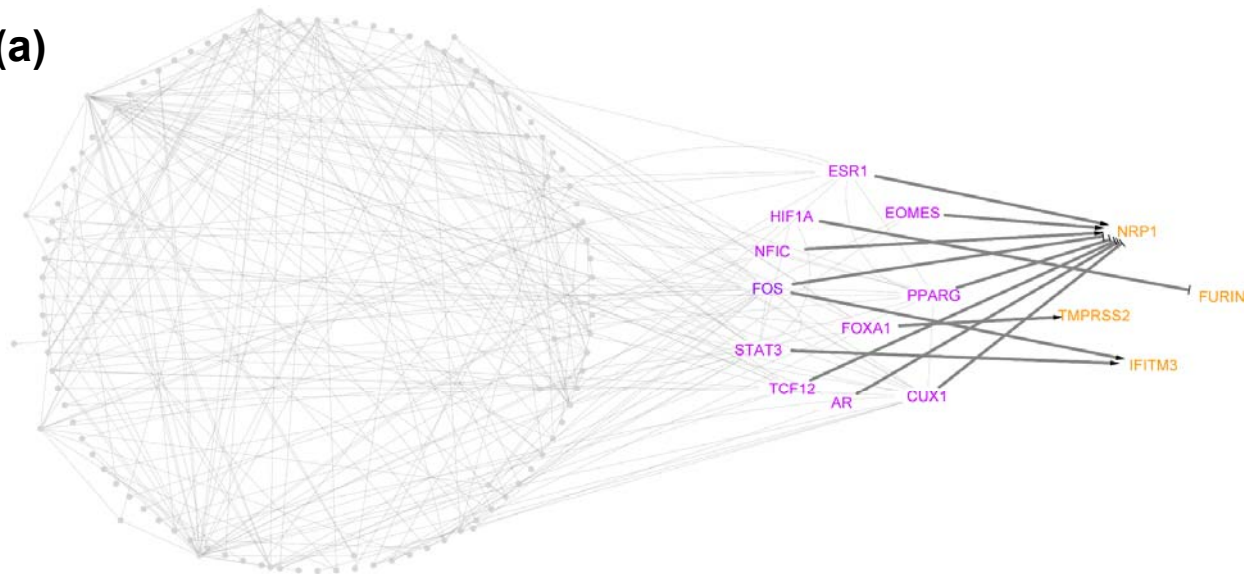

**(b)**

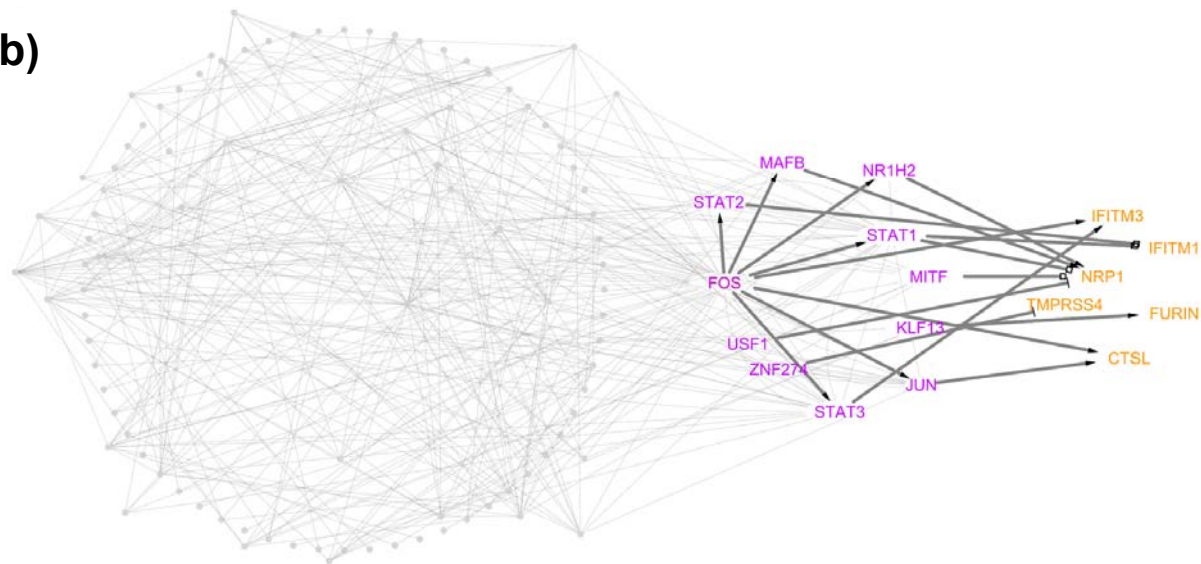

Supplement: Supplementary file 1 [file viruses-14-00837-s001.zip › Figure S5.pdf]

(a)

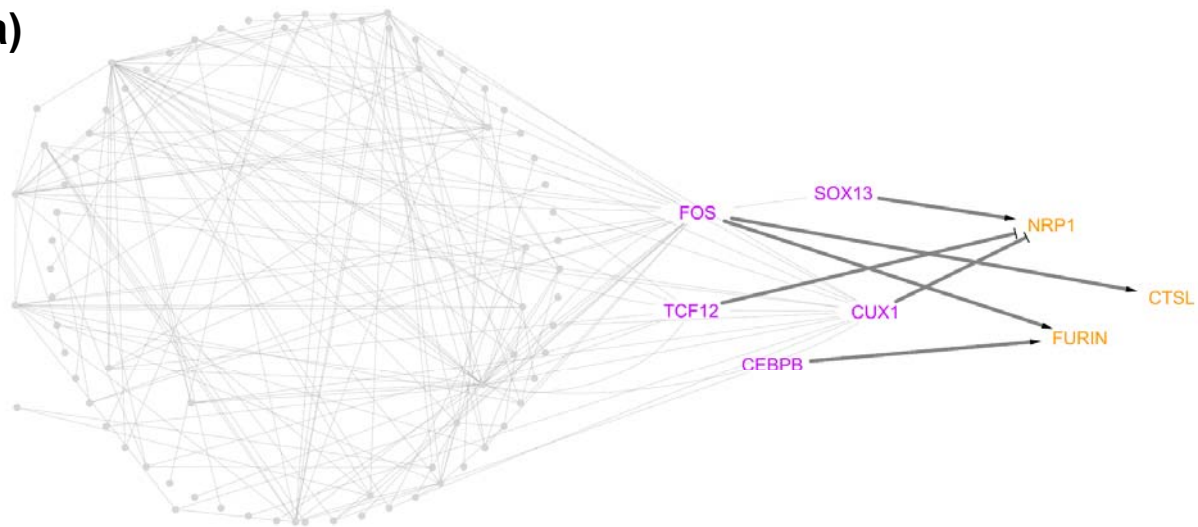

(b)

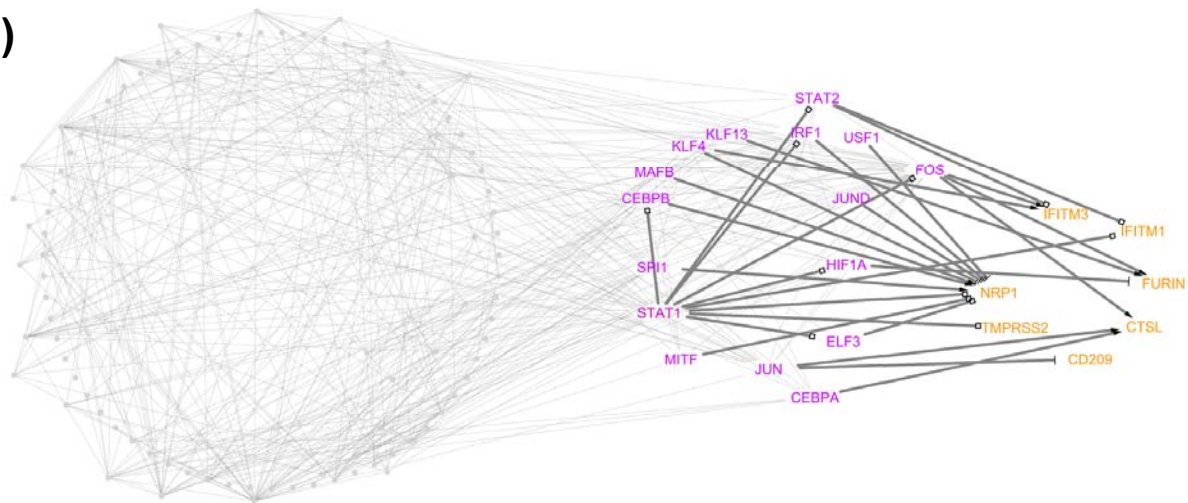

Supplement: Supplementary file 1 [file viruses-14-00837-s001.zip › Figure S6.pdf]

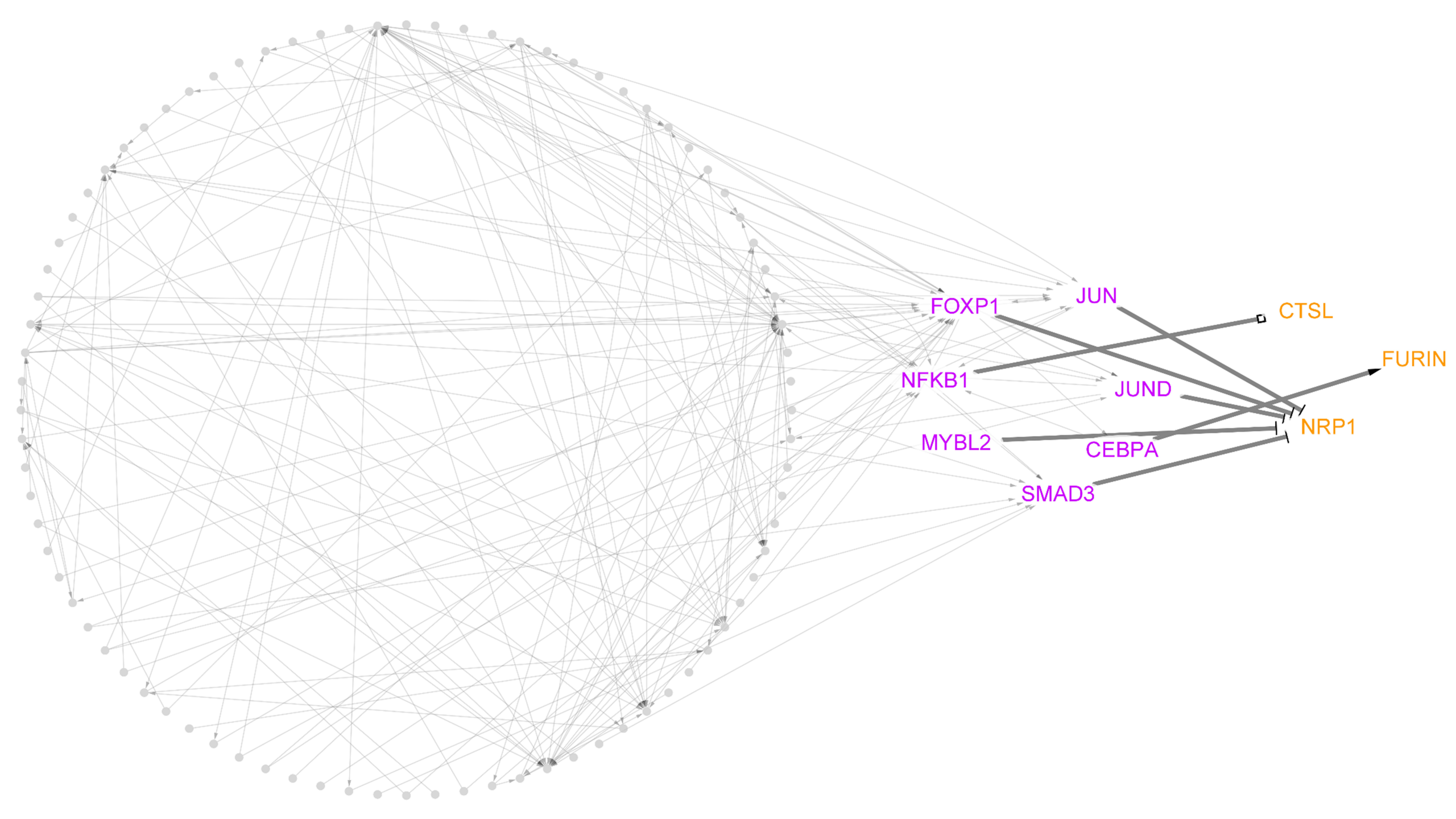

Supplement: Supplementary file 1 [file viruses-14-00837-s001.zip › Figure S7.pdf]

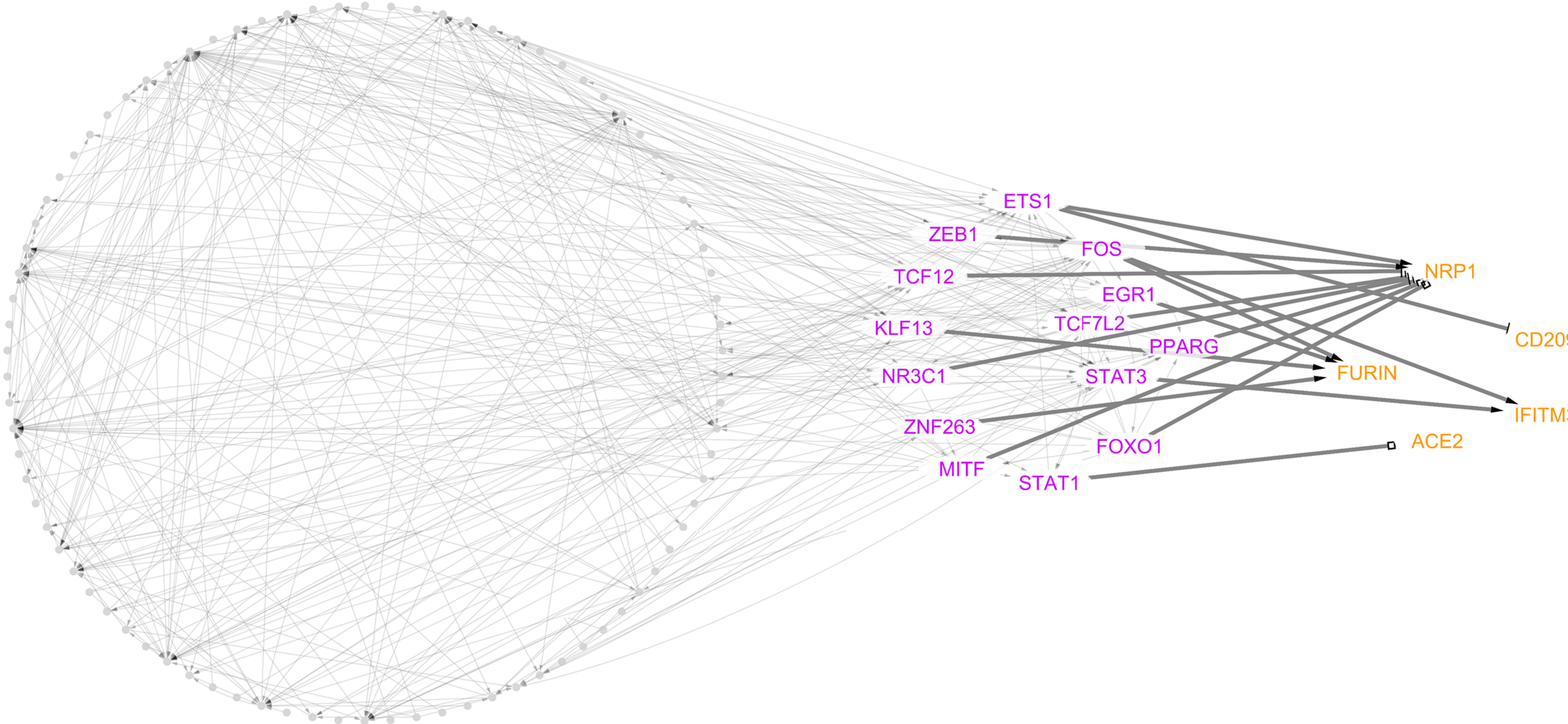

Supplement: Supplementary file 1 [file viruses-14-00837-s001.zip › Figure S8.pdf]

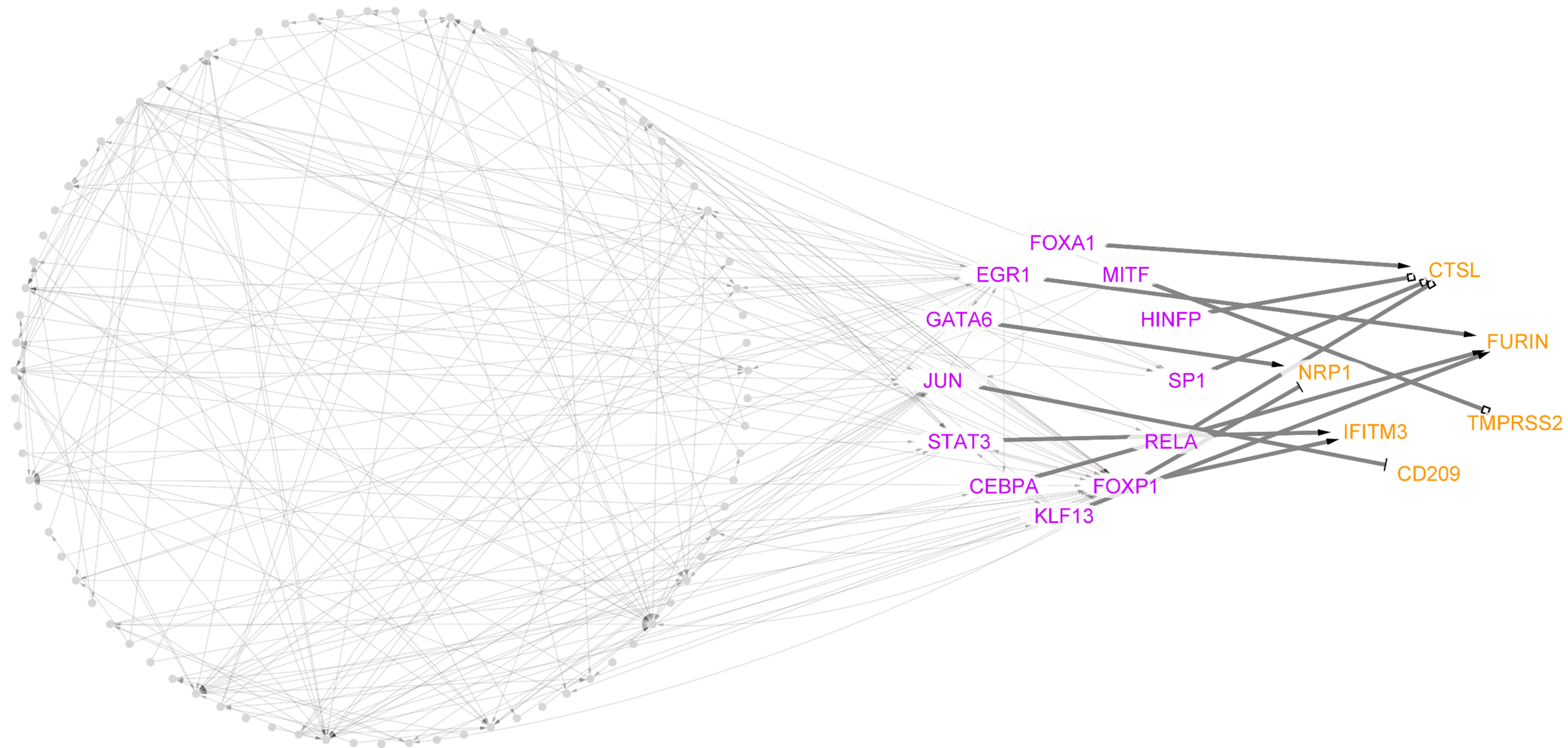

Supplement: Supplementary file 1 [file viruses-14-00837-s001.zip › Figure S9.pdf]
