# Supplementary material for: Identification of Transcription Factors Regulating SARS-CoV-2 Tropism Factor Expression by Inferring Cell-Type-Specific Transcriptional Regulatory Networks in Human Lungs
Source: Viruses. 2022 Apr 17;14(4):837. doi: 10.3390/v14040837 (PMC9026071; doi:10.3390/v14040837)
Supplement: Supplementary file 1 [file Figure S16.pdf › Figure S16.pdf]

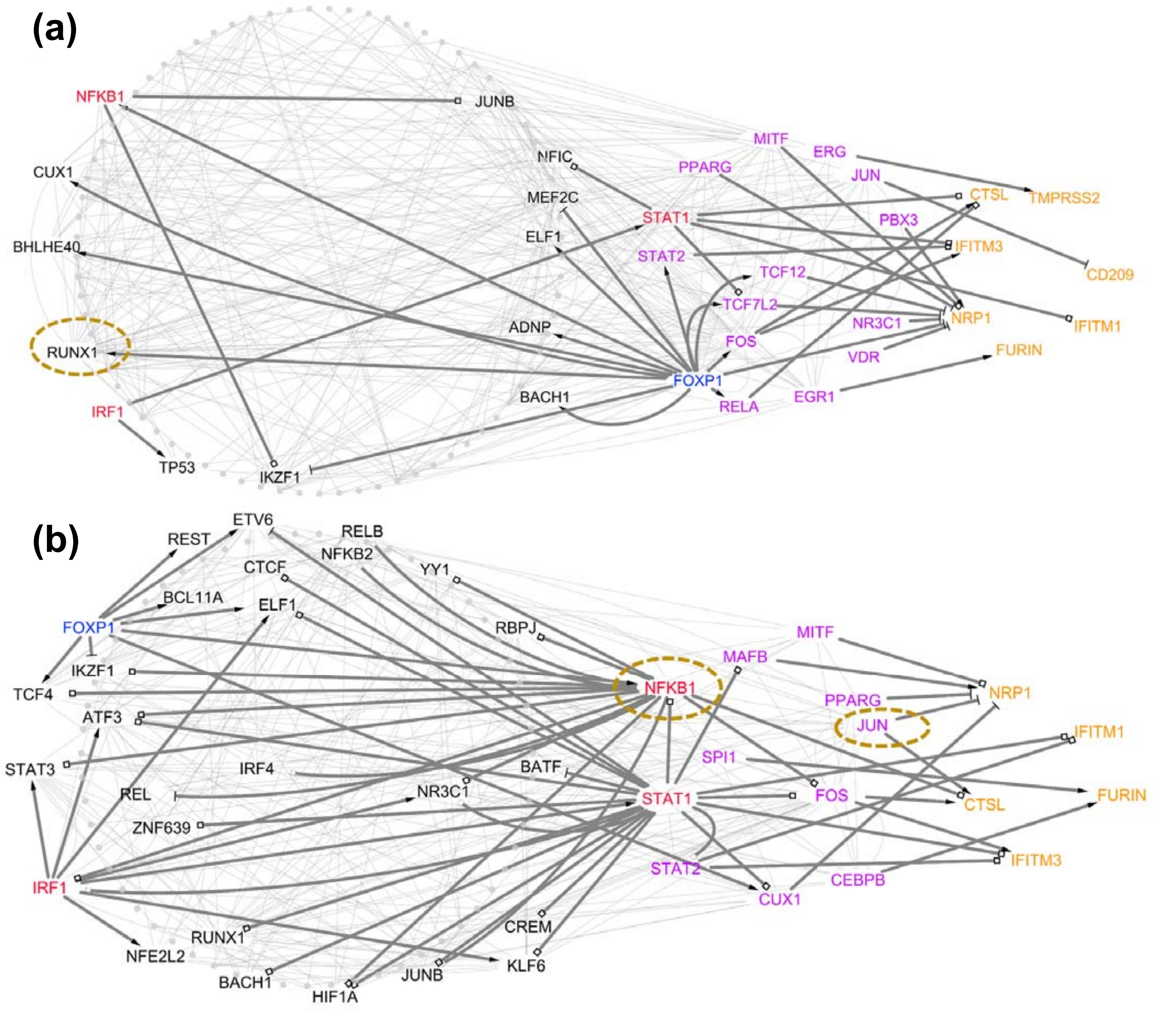

(a)
NFKB1
JUNB
CUX1
NFIC
MITF
ERG
PPARG
JUN
MEF2C
CTSL
TMPRSS2
STAT1
PBX3
BHLHE40
ELF1
IFITM3
STAT2
CD209
TCF12
TCF7L2
NRP1
NR3C1
IFITM1
ADNP
FOS
RUNX1
VDR
FURIN
FOXP1
BACH1
EGR1
RELA
IRF1
TP53
IKZF1
(b)
ETV6
REST
RELB
NFKB2
CTCF
YY1
BCL11A
ELF1
FOXP1
RBPJ
MITF
MAFB
IKZF1
NFKB1
NRP1
TCF4
PPARG
ATF3
JUN
IFITM1
SPI1
STAT3
IRF4
BATF
REL
NR3C1
STAT1
FOS
FURIN
CTSL
ZNF639
IFITM3
IRF1
STAT2
CEBPB
CUX1
CREM
RUNX1
NFE2L2
KLF6
BACH1
JUNB
HIF1A
